# FAST, a method based on split-GFP for the detection in solution of proteins synthesized in cell-free expression systems

**DOI:** 10.1038/s41598-024-58588-5

**Published:** 2024-04-05

**Authors:** Thuy Duong Pham, Chiara Poletti, Therese Manuela Nloh Tientcheu, Massimiliano Cuccioloni, Roberto Spurio, Attilio Fabbretti, Pohl Milon, Anna Maria Giuliodori

**Affiliations:** 1 Laboratory of Genetics of Microorganisms and Microbial Biotechnology, School of Biosciences and Veterinary Medicine, University of Camerino, 62032 Camerino, MC Italy; 2 Laboratory of Biomolecules, Faculty of Health Sciences, Universidad Peruana de Ciencias Aplicadas (UPC), Lima, Peru

**Keywords:** Split-GFP, Protein synthesis, CFPS, Fluorescence, CspA, Biological techniques, Molecular biology

## Abstract

Cell-free protein synthesis (CFPS) systems offer a versatile platform for a wide range of applications. However, the traditional methods for detecting proteins synthesized in CFPS, such as radioactive labeling, fluorescent tagging, or electrophoretic separation, may be impractical, due to environmental hazards, high costs, technical complexity, and time consuming procedures. These limitations underscore the need for new approaches that streamline the detection process, facilitating broader application of CFPS. By harnessing the reassembly capabilities of two GFP fragments—specifically, the GFP1-10 and GFP11 fragments—we have crafted a method that simplifies the detection of in vitro synthesized proteins called FAST (Fluorescent Assembly of Split-GFP for Translation Tests). FAST relies on the fusion of the small tag GFP11 to virtually any gene to be expressed in CFPS. The in vitro synthesized protein:GFP11 can be rapidly detected in solution upon interaction with an enhanced GFP1-10 fused to the Maltose Binding Protein (MBP:GFP1-10). This interaction produces a fluorescent signal detectable with standard fluorescence readers, thereby indicating successful protein synthesis. Furthermore, if required, detection can be coupled with the purification of the fluorescent complex using standardized MBP affinity chromatography. The method's versatility was demonstrated by fusing GFP11 to four distinct *E. coli* genes and analyzing the resulting protein synthesis in both a homemade and a commercial *E. coli* CFPS system. Our experiments confirmed that the FAST method offers a direct correlation between the fluorescent signal and the amount of synthesized protein:GFP11 fusion, achieving a sensitivity threshold of 8 ± 2 pmol of polypeptide, with fluorescence plateauing after 4 h. Additionally, FAST enables the investigation of translation inhibition by antibiotics in a dose-dependent manner. In conclusion, FAST is a new method that permits the rapid, efficient, and non-hazardous detection of protein synthesized within CFPS systems and, at the same time, the purification of the target protein.

## Introduction

Cell-free protein synthesis systems (CFPS) exploit the cell's transcriptional/translational apparatus to obtain the desired protein product. Used since unveiling the genetic code^1^ it is now applied in synthetic biology studies (for a review see^2,3^) or in basic science to investigate cis- and trans-elements that regulate gene expression at the translational level^4,5^. On the commercial side, CFPS are used to produce otherwise insoluble or toxic proteins, to perform high-throughput screening of therapeutic proteins (for a review see^3,6^), and to characterize the molecular mechanism of lead molecules with antibiotic properties^7^.

CFPS systems can be produced using extracts prepared from both bacteria and archaea, as well as from eukaryotic cells, and they can be either prepared in the laboratory or purchased from biotech companies in the form of commercial kits^6^. CFPS can couple mRNA transcription and translation or cover only the translation system. The synthesized protein can be detected/captured/quantified using different methods depending on the desired applications. Hence, the protein can be captured or isolated by affinity, detected by enzymatic activity assays, identified by mass spectrometry, Western blot^8^ or quantified by ELISA or radioactive labelling of specific aminoacids^7^. Additionally, luminescence or  fluorescence-based assays can be applied in CFPSs. Invitrogen Lumio™ Green Detection-Experienced (Thermofisher) and the FluoroTect™ GreenLys (Promega) can detect in vitro expression of proteins by making use of fluorescent dyes that bind the engineered reporter polypeptide. Both commercial systems require electrophoretic separation to observe the fluorescently tagged protein.

One possible method for studying the efficiency of CFPS systems is based on the use of the reporter Green Fluorescent Protein (GFP). The GFP has a peculiar structure characterized by a β-barrel consisting of 11 strands with two short helices at the ends and a long helix that runs through the β-barrel that contributes to build the fluorogenic core^9^. Fluorescence emission is achieved by amino acids S65-Y66-G67 which form a heterocyclic ring (p-hydroxybenzylidene-imidazolidone) in a process of cyclization and oxidation^9^. This chromophore maturation process has a slow rate compared to the folding kinetics of the GFP proteins^10^. The superfolder GFP (sfGFP) variant^11^ is a protein with optimized folding kinetic that has been successfully used in coupled prokaryotic transcription/translation systems (see references in^3,6^). Also the Emerald GFP (emGFP)^12^ has been employed for real-time monitoring of protein synthesis in a coupled transcription/translation system^13^.

In 2005, Stephanie Cabantous et al. have demonstrated that a variant of the sfGFP can be reassembled from two separate fragments, in a complementation system called split-GFP. The large polypeptide, called GFP1-10, contains the first ten β-strands of the GFP, whereas the small fragment, called GFP11, consisting of the last 16 amino acids of the protein, corresponds to the last GFP β-strand. GFP 1–10 can interact with the small GFP11 fragment, fused to the C-terminus of other proteins, when this fusion is overproduced in vivo and also when expressed in a crude *E. coli* extract. The reassembled GFP protein is fully functional and emits fluorescence^14^.

The split-GFP system requires a minimum of two steps to regain activity: first, the association of the large and small GFP fragments, which occurs at a rate of 0.0042 ± 0.0002 µM^−1^ min^−1^^10^. Only after association of both fragments, the GFP complex can slowly rearrange into a matured protein that is capable of fluorescence emission (K_mat_ = 0.016 ± 0.001 min^−1^^10^). Also other split-GFP variants show a similar behavior, with comparable association and maturation rates^10^. Notably, the GFP1-10 fragment is preferentially found as a dimer at high concentration when purified as a non-fusion protein, but only its monomeric form is able to assemble with the GFP11 fragment^10^. In addition, the GFP1-10 fragment is partly insoluble and may require purification from inclusion bodies under denaturing conditions^14^.

Split GFP systems have been and continues to be a source of inspiration for numerous applications in various fields, ranging from solubility monitoring, to protein labeling in mammalian cells, subcellular localization, and biochemical studies^15^.

**Figure 1:**
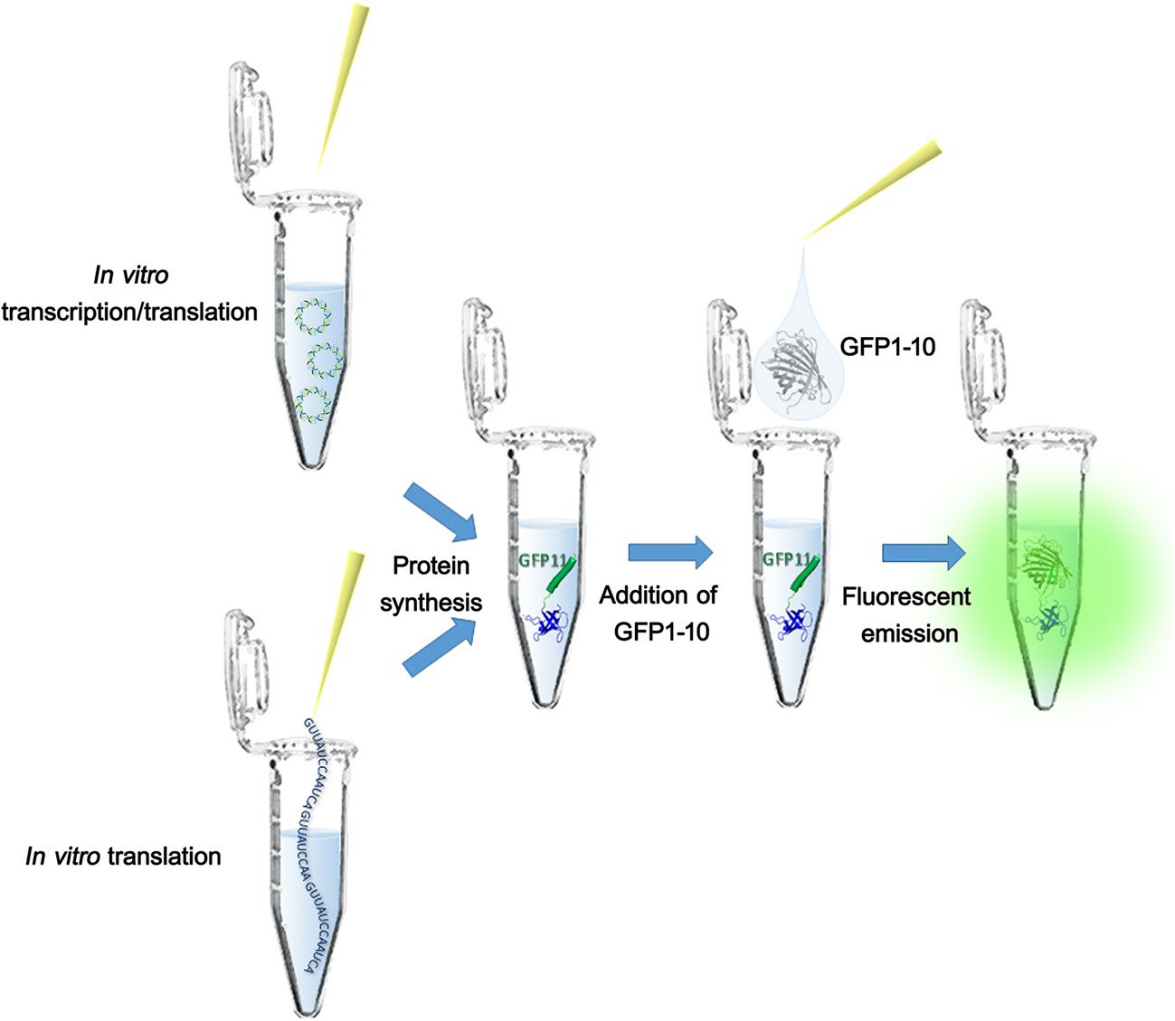
FAST system for monitoring protein synthesis in vitro using fluorescence. The DNA encoding the protein of interest must be fused in-frame with the "GFP11" tag. In the presence of DNA or mRNA produced from this template and the necessary components for protein synthesis, the corresponding fusion gene product will be synthesized. By adding the GFP1-10 fragment, a fluorescence signal proportional to the amount of protein produced will be generated.

In this work we developed an enhanced split GFP system, called FAST (Fluorescent Assembly of Split-GFP for Translation Test) to track the synthesis of virtually any non-fluorescent protein in *E. coli* CFPS systems. The detection of the synthesized protein in the cell-free system is based on the complementation process between the two GFP fragments. Therefore, the DNA coding for the protein of interest is fused in frame with the sequence for the "GFP11" tag through a short flexible linker. When the DNA or the mRNA is placed in a reaction tube that contains all components for protein synthesis, the corresponding fusion protein will be synthesized (Fig. 1). By adding the GFP1-10 fragment at the end of the CFPS reaction, a fluorescence signal develops proportional to the molar concentration of the GFP11-fused product. The sequence coding for the GFP11 tag can be fused to any gene of interest with the advantage of being smaller if compared to the full GFP ("linker + tag" = 26 amino acids). Unlike in vivo protein expression, CFPS assays rely on finite sources of ATP/GTP and amino acids that largely limit protein expression yields. Therefore, shorter reporters result in higher molar product yields if compared to larger fragments.

In an attempt to overcome the limitations concerning the timing of split complementation we decided to develop a new split GFP system based on a fast folder GFP (ffGFP) previously evolved in our laboratory^16^. This protein variant displays good resistance to pH variations, remarkable temperature-resistance (half-life at 85 °C and 90 °C of 197 s and 73 s, respectively), and a refolding kinetics similar to sfGFP (4.16 ± 0.08 10^−1^ s^−1^ vs 5.0 10^−1^ s^−1^). Additionally, we fused the GFP1-10 fragment to the Maltose Binding Protein (MBP), which enhances solubility, prevents aggregation of recombinant proteins in *E. coli*^17^, and is also a useful tag for the purification by affinity chromatography, a feature that could be exploited for the subsequent purification of the in vitro synthesized protein. To our knowledge, none of the current monitoring systems in CFPS allow for the simultaneous detection and purification of the proteins like FAST.

In this study, quantitative detection of protein synthesis levels based on GFP11 tag/MBP:GFP1-10 was validated with various gene fusions of increasing length, using both in house and commercial CFPS prepared from *E. coli* cells.

## Results

### Development of a new split GFP system

**Figure 2:**
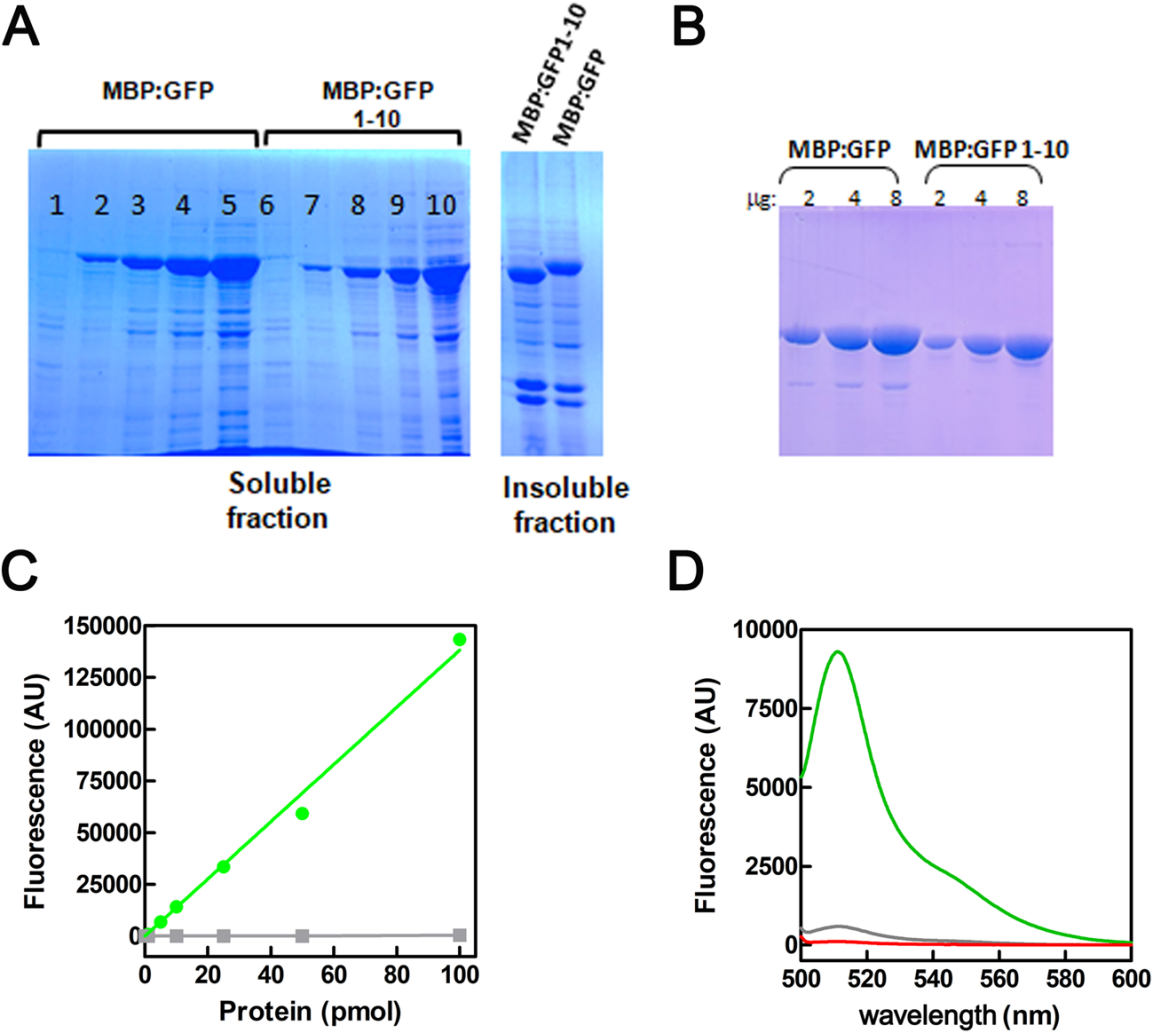
Solubility and fluorescence of MBP:GFP and MBP:GFP1-10. (**A**) Electrophoretic separation by 10% SDS-PAGE of 2, 4, and 8 µL of the soluble fraction of *E. coli* Stellar (crude extracts) containing MBP:GFP (lanes 3–5) or MBP:GFP1-10 (lanes 8–10). Lanes 1 and 6 and 2 and 7 were loaded with 10 µL of total cellular lysate taken before and after IPTG induction, respectively. The insoluble fraction represents the pellet produced after cell disruption and removal of unlysed cells, resuspended in 6 M urea. (**B**) Increasing amounts of the two purified proteins analyzed by 10% SDS-PAGE. The full uncropped gel pictures are shown in the Supplementary file, Fig. S1. (**C**) The fluorescence of 1, 5, 10, 25, 50, and 100 pmol of either MBP:GFP (green) or MBP:GFP1-10 (gray) was measured in 100 μL of Storage buffer using a Black & White Isoplate 96-well Wallac microtiter plate in the FLUOstar Omega reader. Fluorescence emitted by control samples missing the proteins has been subtracted. (**D**) The complementation reaction was conducted in 500 μL of Storage buffer with 500 pmol of MBP:GFP (green), 500 pmol of MBP-GFP1-10 (gray) or 500 pmol of MBP-GFP1-10 + 1 nmole of GFP11 peptide (red). The spectrum was recorded between 500 and 600 nm at room temperature with a Shimadzu RF-5301PC (Shimadzu, Japan) (slit = 5, PMT = 950).

To develop a soluble and easily purifiable GFP1-10 fragment, we transferred through molecular cloning the ffGFP, which exhibits thermo-tolerant properties and a higher refolding rate compared to the parent GFP (ASV) protein^16^ into the pMAL-c5x vector (New England BioLabs Inc.). As a result of this cloning procedure, two N-terminal fusions were produced: (i) the fusion between MBP and the full-length GFP and (ii) the fusion between MBP and the GFP1-10 fragment. Both recombinant proteins expressed in *E.coli* are present in the soluble fraction (Fig. 2A) and their purification (Fig. 2B) yielded approximately ~ 15 mg/g of cells. The fluorescence-emitting capacity of the full-length GFP is not affected by the fusion with MBP (Fig. 2C, green), while the MBP:GFP1-10 protein shows no activity, as it lacks the last strand (Fig. 2C, D, gray). However, MBP:GFP1-10 is unable to form a functional complex capable of emitting fluorescence also when mixed with a synthetic GFP11 peptide corresponding to the missing β-strand (Fig. 2D, red trace).

**Figure 3:**
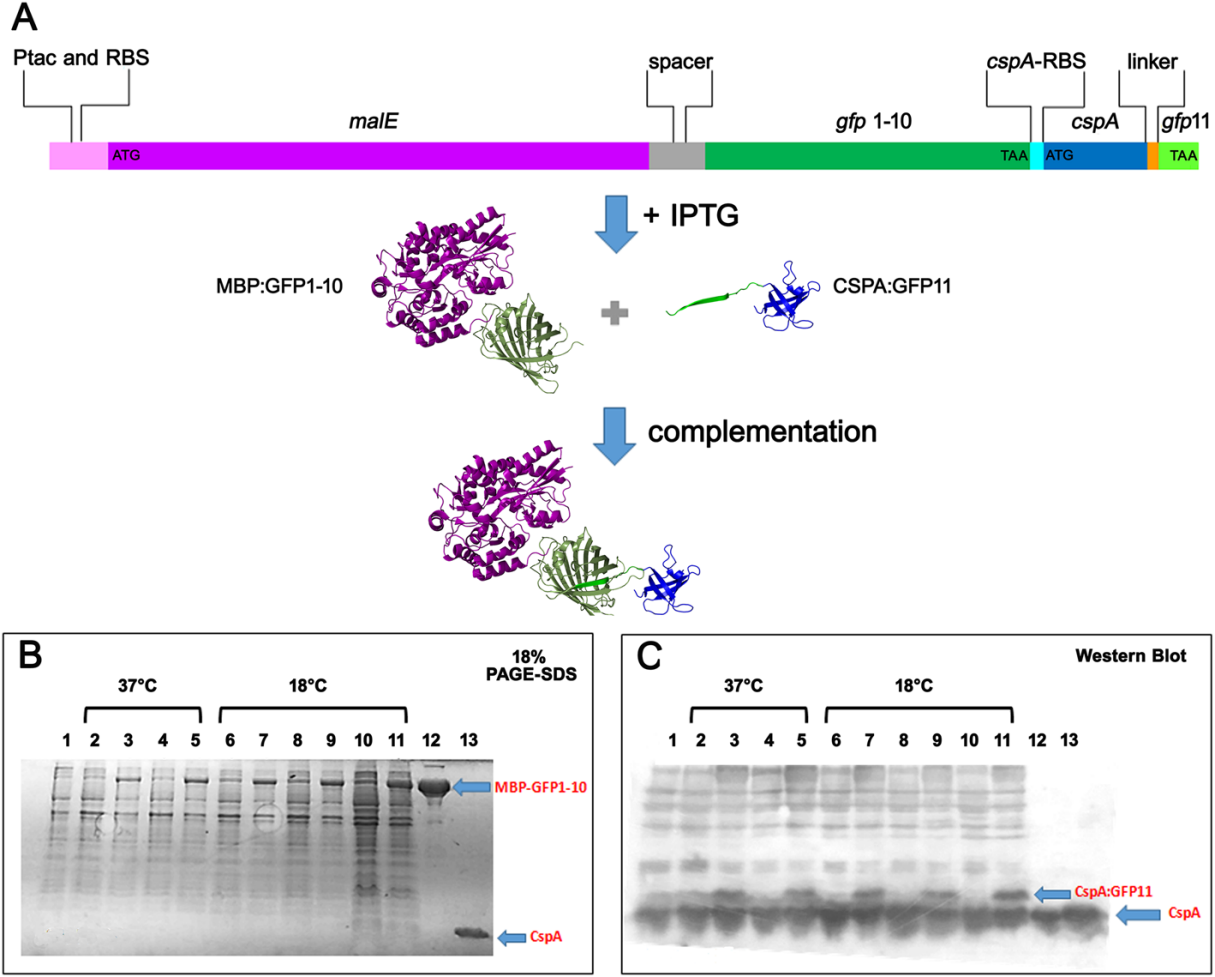
Schematic representation of the bicistronic construct inserted into the pMAL vector and analysis of fragments expression (**A**) The construct was cloned downstream of the *malE* gene (purple), controlled by the Ptac promoter (pink) and by the adjacent RBS (pink). The *gfp1-10* fragment (dark green) was cloned immediately downstream the spacer present in the vector, which contains the cleavage site for the Xa factor (gray). Downstream the stop codon of *gfp1-10*, a 30 bp fragment (cyan) containing the SD of the *cspA* gene is inserted, followed by the *cspA* gene (blue) separated from the *gfp11* fragment (light green) by a linker (orange). Transcription from the Ptac promoter induced by IPTG produces a bicistronic mRNA, the translation of which leads to the production of the two fusion proteins, MBP:GFP1-10 and CSPA:GFP11 and, potentially, to complementation. (**B**) Analysis of MBP:GFP1-10 expression by 18% SDS-PAGE and (**C**) Analysis of CSPA:GFP11 expression by Western blot. Protein synthesis was induced in *E. coli* Stellar cells containing the bicistronic construct. The cell culture was divided into two portions, one treated with IPTG (lanes 3,5,7,9,11) and the other untreated (lanes 2,4,6,8,10). Samples were taken at 37 °C at 0 (lane 1), 40 (lanes 2, 3) and 80 (lanes 4 and 5) minutes after IPTG addition. The two cultures were then transferred to 18 °C, and additional samples were taken at 30 (lanes 6 and 7), 60 (lanes 8 and 9) minutes, and after overnight incubation (lanes 10 and 11) from the time of temperature shift. The bands corresponding to the target proteins are indicated by an arrow. The full uncropped gel and westen blot pictures are shown in Fig. S3.

To develop and select a functional GFP-split system, we created the bicistronic construct depicted in Fig. 3A. In this construct *gfp1-10* is cloned downstream of the *malE* gene, which encodes the MBP protein. *Gfp1-10* sequence is followed by the Ribosome Binding Site (RBS) of the cold-shock gene *cspA,* which is a soluble small β-barrel protein highly abundant in *E. coli* during cold stress as well as at 37 °C during the initial phases of cell growth^18^. The expression of CspA is mainly controlled at the translational level^4,5^, and the 30-base pair of the cspA’s RBS cloned in the pMAL-c5x plasmid contains both the Shine-Dalgarno (SD) sequence and the AT-rich region preceding it, which is a putative binding site for the ribosomal protein S1^4^. This RBS is followed by the *cspA* coding sequence fused at the C-terminus to a linker encoding the sequence (Gly-Gly-Gly-Ser)2, and then by the *gfp11* fragment. The expression of the bicistronic mRNA is controlled by the Ptac promoter present in the pMAL plasmid upstream of the *malE* gene (Fig. 3A). In response to IPTG, the bicistronic mRNA accumulates in the cells and is translated, producing the two fusion proteins MBP:GFP1-10 and CSPA:GFP11. Regarding the latter fragment, its nucleotide sequence has been modified compared to the *gfp11* sequence of ffGFP, both to optimize its translation in *E. coli* and to introduce an amino acid change (Val → Asp) that, based on the protein conformation, might favor the complementation reaction. The nucleotide and amino acid sequence of the *cspA:gfp11* fusion, with the mentioned changes, are shown in Fig. S2.

The expression of the two translational fusions from the pMAL:GFP1-10-CSPA:GFP11 construct was monitored at various time points after induction, both at 37 °C and after transferring the samples to low temperature (Fig. 3B,C), to possibly improve the translation of the cold-shock *cspA*:gfp11 fusion. Both proteins are synthesized as early as 40 min after IPTG addition at 37 °C, they continue to accumulate at later time points and are present even after overnight incubation at 18 °C. Semi-quantitative analysis of the bands corresponding to the two products of the construct (MBP:GFP1-10 and CSPA:GFP11, Fig. 3B,C) demonstrates that the system based on the use of the *cspA*'s RBS for the expression of the second cistron could be useful to achieve the expression of two proteins in *E. coli* at near equimolar levels. However, under any of the tested conditions, we did not observe the development of fluorescence, confirming that the MBP:GFP1-10 fusion is unable to complement with the GFP11 fragment, also in vivo.

To make the split system functional, we introduced three amino acid substitutions into the GFP1-10 sequence through site-directed mutagenesis, namely S30R, which plays a major role in stabilizing the β-barrel^11^, and S205T and A206V, chosen among those present in the

GFP 1–10 OPT^14^. The construct containing these amino acid substitutions (named pMAL:GFP1-10s1-CSPA:GFP11) made the transformed *E. coli* cells to glow. Comparison between the GFP1-10 and GFP1-10s1 sequences is shown in Fig. S2. Interestingly, in the absence of IPTG, fluorescence developed only when the plates were first incubated at 37 °C and then transferred for at least 24 h to temperatures ≤ 20 °C, unlike cells transformed with the MBP:GFP construct, which became fluorescent after overnight incubation at 37 °C (Fig. S4).

### Enhanced GFP1-10 by in vitro evolution

To improve the fluorescence intensity of the complementation system, we generated a random mutant library of the GFP1-10s1 sequence using error-prone PCR (epPCR), which induces erroneous nucleotide incorporation during DNA amplification. Under the conditions used, the frequency of substitutions averaged around 6 base pairs per kilobase of DNA. The mutants were re-cloned into the bicistronic construct and selected in vivo for their ability to emit fluorescence upon complementation.

**Figure 4:**
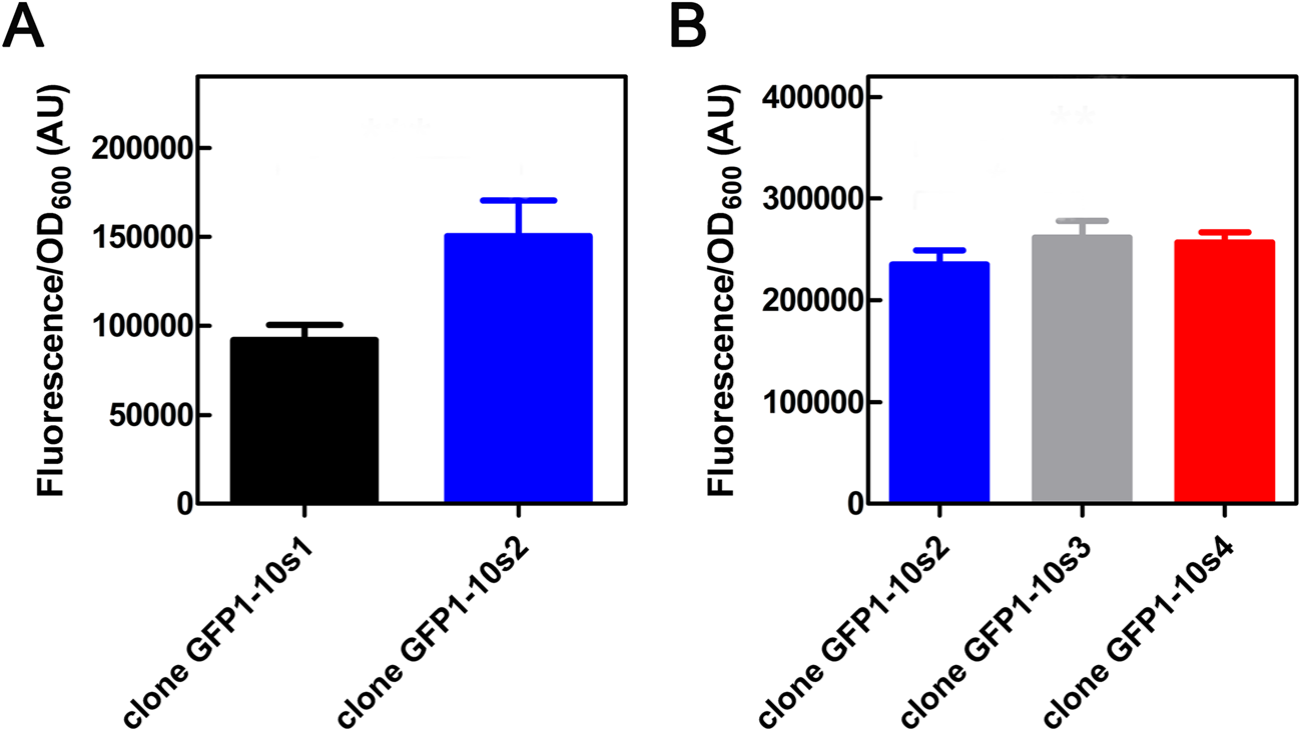
In vivo complementation assay of GFP1-10 variants generated by error-prone PCR. (**A**) Histograms comparing the fluorescence emitted by the original construct (GFP1-10s1) with clone GFP1-10s2 obtained after the first round of epPCR and subsequent construction of a library of random mutants and screening. The fluorescence intensity was normalized to the corresponding OD_600_ of the culture. A Student's *t*-test on these data produces a *P* value = 0.0004. (**B**) Comparison between GFP1-10s2, and its evolved variants GFP1-10s3 and GFP1-10s4. Error bars indicate the standard error calculated from triplicate measurements in two independent experiments. Background fluorescence of the culture medium was subtracted. The fluorescence intensity was normalized to the corresponding OD_600_ of the culture. Data were analyzed by one-way Anova test, which computed a *P* value = 0.0103.

Approximately 16% of the transformants obtained from the first mutant library were fluorescent. This result suggests that the majority of mutations had a negative impact on complementation or fluorescence emission. Among these fluorescent clones, only one, denominated pMAL:GFP1-10s2-CSPA:GFP11, showed higher fluorescence than the starting construct pMAL:GFP1-10s1-CSPA:GFP11 (Fig. 4A). The DNA of GFP1-10s2, purified and sequenced, was then subjected to a second round of random mutagenesis, followed by library preparation and screening. At the end of the second screening, approximately 30% of colonies were fluorescent. In this case, two clones named pMAL:GFP1-10s3-CSPA:GFP11 and pMAL:GFP1-10s4-CSPA:GFP11 with a slightly higher fluorescence than pMAL:GFP1-10s2-CSPA:GFP11 were identified (Fig. 4B) and sequenced. The two rounds of random mutagenesis of the GFP1-10 fragment improved the overall efficiency of the split system by approximately 80%, estimated by comparing the fluorescence of pMAL:GFP1-10(s3/4)-CSPA:GFP11 to pMAL:GFP1-10s1-CSPA:GFP11.

GFP1-10s2 contains the N149K substitution, GFP1-10s3 carries the N149K and Y151C substitutions, while GFP1-10s4 has an identical amino acid sequence to GFP1-10s2 but carries a silent mutation near the other two mentioned mutations, which changes the codon GTA to GTT, both encoding for Val (Figs. S5 and S6). The positions of the mutations in the three-dimensional structure of GFP, considering the other mutations introduced through site-directed mutagenesis, are shown in Fig. S7. It is worth noting that the N149K substitution is present in eGFP and was found to be associated with an enhancement of the green color^19^. As for the nature of mutations found in GFPs3 and GFPs4, the comparison between the variants was independently repeated three times, each time in triplicate, and the differences between the means, despite being small, were statistically significant. However, the limited improvement of these variants in terms of efficiency, along with the presence of the silent mutation in s4, leads us to two interpretations: that either the difference in fluorescence between GFP1-10_S2_, GFP1-10_S3_, and GFP1-10_S4_ is an artifact or that mutations in s3 and s4 variants may influence the protein expression level rather than the structural/functional properties of GFP.

**Figure 5:**
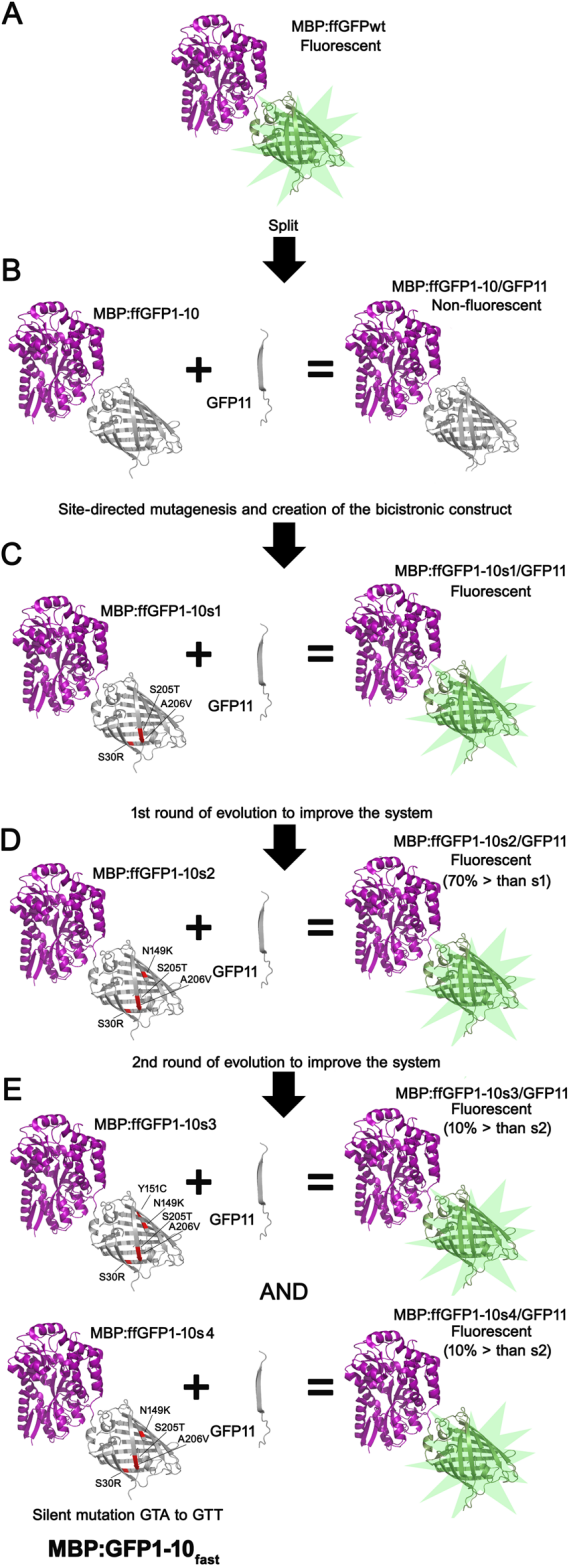
Schematic representation of the sequential steps to obtain MBP:GFP1-10_fast_ from ffGFP. (**A**) The fast-folding variant GFP (ffGFP) was fused with GFP, and it was ascertained that the fusion retained the same properties as the original ffGFP; (**B**) Production of the MBP:GFP1-10 fragment, which demonstrated an inability to complement with GFP11; (**C**) After introducing the amino acid substitutions indicated in MBP:GFP1-10s1 through site-directed mutagenesis, in vivo fluorescence was achieved within our split-GFP system; (**D**) The first round of system evolution was conducted through epPCR, resulting in the selection of a MBP:GFP1-10s2 variant exhibiting an approximate 70% increase in fluorescence; (**E**) The second round of evolution was carried out via ePCR, leading to the identification and selection of two variants displaying an additional 10% enhancement in fluorescent signal. The preferred variant, designated MBP:GFP1-10s4, was ultimately named MBP:GFP1-10_fast_.

The GFP1-10s4 sequence was chosen for further steps in this study, and was named MBP:GFP1-10_fast_. All the steps that led from the initial fast-folding GFP to MBP:GFP1-10_fast_, along with the properties of the various intermediate split-GFP proteins, are summarized in Fig. 5. At the end of the process, the two GFP fragments were cloned into two different expression vectors: the MBP:GFP1-10_fast_ sequence remained in the pMAL-X5 plasmid, while the CSPA:GFP11 fragment was removed and cloned into the pETM11 plasmid (EMBO). When *E. coli* BL21 (DE3) cells were co-transformed with the two plasmids (pMAL:GFP1-10_fast_ and pET11-CSPA:GFP11), fluorescence increased over time (Fig. S8A) upon protein induction (Fig. S8B). As expected, no increase in fluorescence was observed even three hours after IPTG induction (Fig. S8A) when the two proteins were separately overproduced in *E. coli* (Fig. S8C). It should be noted that the level of simultaneous overproduction of the two proteins in the analyzed samples is high but variable, most likely because the copy number of the two plasmids used cannot be controlled as they share the same regulon.

### FAST performance in CFPS

After documenting complementation between the two fragments, we proceeded to perform the in vitro CFPS (cell-free protein synthesis) assays. For this purpose, the *gfp11* tag was cloned into the pUT7cspA vector, which contains the entire *cspA*, including the 5' and 3' untranslated regions (UTR) of the gene^4^. The sequence of the resulting construct is shown in Fig. S10. Subsequently, both pUT7cspA and pUT7cspA:gfp11 plasmids were transcribed in vitro using the T7 RNA polymerase to produce the mRNAs to be used in the cell-free translation system. To obtain complementation in vitro, also MBP:GFP1-10_fast_ was overexpressed and purified from pMAL:GFP1-10_fast_.

In the first set of experiments, the translation reaction was conducted using an in-house crude S30 run-off extract prepared from *E. coli* cells^7,20^. The quantity of synthesized proteins was first estimated by measuring the incorporation of [^3^H]-Phe in each reaction tube. The same experiment was then conducted under identical conditions but without the radioactive amino acid. At the end of the incubation, an excess of MBP:GFP1-10_fast_ was added to each tube, and the reaction was further incubated at 25 °C for at least 2 h before measuring fluorescence.

**Figure 6:**
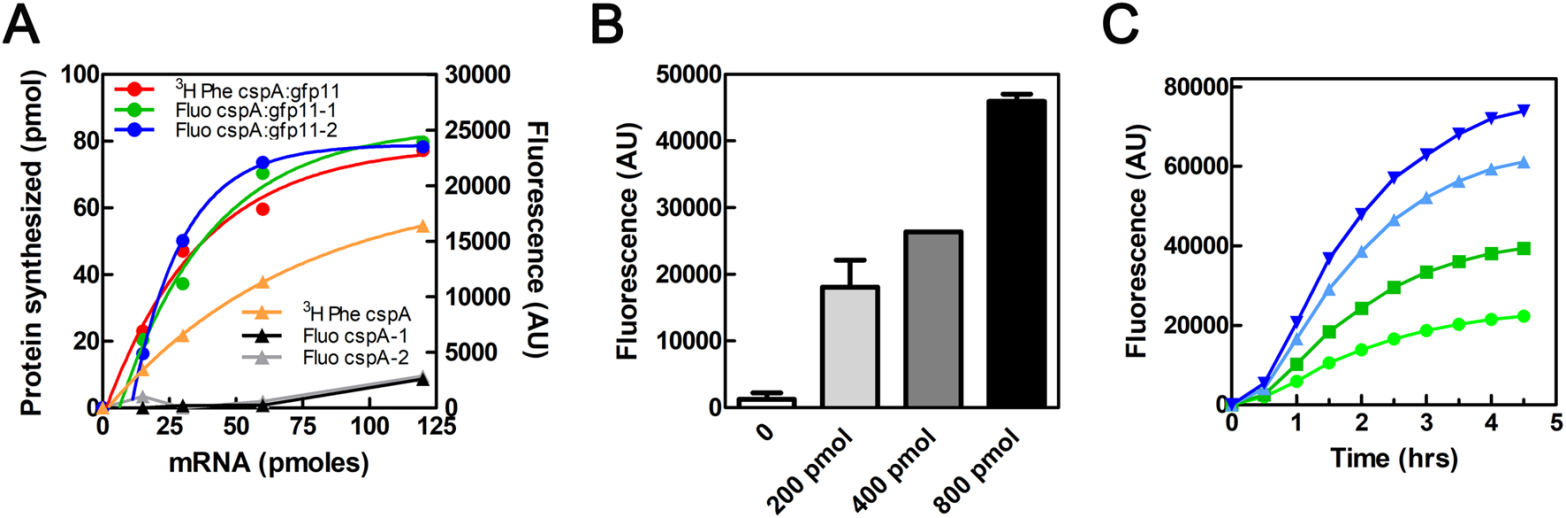
CSPA:GFP11 can be translated in vitro and can quantitatively complement MBP:GFP1-10_fast_. (**A**) Translation of *cspA:gfp11* mRNAs and *cspA* mRNA was performed at 37 °C for 30 min under the conditions described in Materials and Methods using an *E. coli* S30 run-off extract. Protein synthesis was monitored by [^3^H]-Phe incorporation (red trace for *cspA:gfp11* mRNA, orange trace for *cspA* mRNA) or by taking advantage of complementation between the GFP fragments (blue and green traces for *cspA:gfpA11* mRNA; gray and black traces for *cspA* mRNA). In the latter case, after the 37 °C incubation, 800 pmol of MBP:GFP1-10_fast_ were added to each reaction tube, which was further incubated at 25 °C for 2 h before measuring fluorescence at 520 nm. Fluorescence measured in control tubes that did not contain mRNA was subtracted. The data points were fitted using the equation Y = A*(1 − exp(− K*(X − X0))) for X > X0. (**B**) Emitted fluorescence resulting from the addition of the indicated amounts of MBP:GFP1-10_fast_ to the *cspA:gfp11* mRNA-containing CFPS reaction. The interaction between the two GFP fragments was performed at 25 °C for 2 h. Error bars represent the standard error calculated from triplicates. (**C**) Increase in fluorescence as a function of increasing time upon the addition of MBP:GFP1-10_fast_ to in vitro protein synthesis reactions monitored at 15 (light green), 30 (green), 60 (light blue), and 120 (blue) µM of *cspA:gfp11* mRNA. In all experiments, fluorescence measured in control tubes that did not contain mRNA was subtracted.

As shown in Fig. 6A, both *cspA:gfp11* mRNA and *cspA* mRNA produced satisfactory incorporation of radioactive phenylalanine (converted into corresponding pmoles of their respective proteins). The fluorescence measured with *cspA:gfp11* mRNA plotted a curve with a trend similar to that obtained with [^3^H]-Phe, indicating successful translation and split-GFP complementation. The synthesis level of CspA is lower than that of CspA:gfp11, ranging between 45 and 60% of the latter depending on the amount of mRNA added to the translation system. However, if we compare the fluorescence signal obtained with equal amounts of expressed proteins (e.g. 25 pmol of CspA and CspA:GFP11, or 50 pmol of CspA and CspA:GFP11), the fluorescence developed with *cspA:gfp11* is significantly higher than that obtained with the control *cspA* mRNA, as expected. By fitting the experimental data with an exponential curve, it was estimated that the smallest amount of protein detectable with FAST under these conditions is 8 ± 2 pmol, equivalent to approximately 0.13 ± 0.03 µM. The optimal amount of MBP:GFP1-10_fast_ to be added for the detection of the synthesized product was ~ 800 pmol/60 µL reaction (Fig. 6B). Fluorescence resulting from the interaction between MBP:GFP1-10_fast_ and CSPA:GFP11 started to develop within half an hour after the addition of MBP:GFP1-10_fast_, and it steadily increased over the following 1.5 h. Then, the fluorescence increase reached a plateau after approximately 4 h. This trend was consistent across the various concentrations analyzed (see Fig. 6C).

The experiment shown in Fig. 6C shows that after 2–2.5 h of complementation the fluorescence signal is ~ 70–80% of the that achieved at plateau after 4.5-h incubation. Therefore, we believe that an incubation time of 2–2.5 h is an excellent compromise to save time and to get a reliable and quantifiable fluorescence signal.

### Kinetics of association and stability of the MBP:GFP1-10_fast_:GFP11 complex

**Figure 7:**
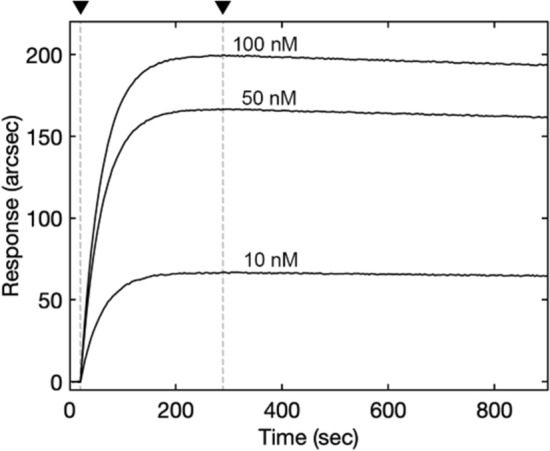
Biosensor study of GFP1-10_fast_ and GFP11 association. Representative superimposition of sensor traces obtained upon addition of diferent concentrations of soluble GFP11 to surface-blocked MBP:GFP1-10_fast_. The dissociation is started with a single wash with fresh buffer. The beginning of association and dissociation events are evidenced (▼).

Previous kinetic studies have shown that the split fragments are able to form a stable, native-like GFP complex and that the dissociation of the noncovalently-bound strand is rate limiting^10,21^. To investigate the interaction between MBP:GFP1-10_fast_ and GFP11 we carried out a standard biosensor assay under the experimental conditions described in Materials and Methods (Fig. 7). MBP:GFP1-10_fast_ showed a strong affinity for the GFP11 peptide, with an equilibrium dissociation constant in the sub-micromolar range (K_D_ < 10^−7^ M). The negligible dissociation phase (k_off_ < 0.0001 s^−1^, the exact value being too low to be calculated accurately) was critical for the high stability of the resulting complex, irrespective of exhibiting slow-binding kinetics (k_on_ = 250 ± 30 M^−1^ s^−1^). The binding kinetics assessed for the MBP:GFP1-10s4 and GFP11 interaction is about 3 times slower than that measured for the variant derived from the sfGFP (k_on_ = 70 ± 0.33 M^−1^ s^−1^ for GFP OPT^10^). As in the case of the split-GFP system relying on sfGFP, once the assembled complexes are formed, they undergo fluorophore maturation, which is also a process with slow kinetics^10,14^.

### Monitoring translational inhibitors with FAST

**Figure 8:**
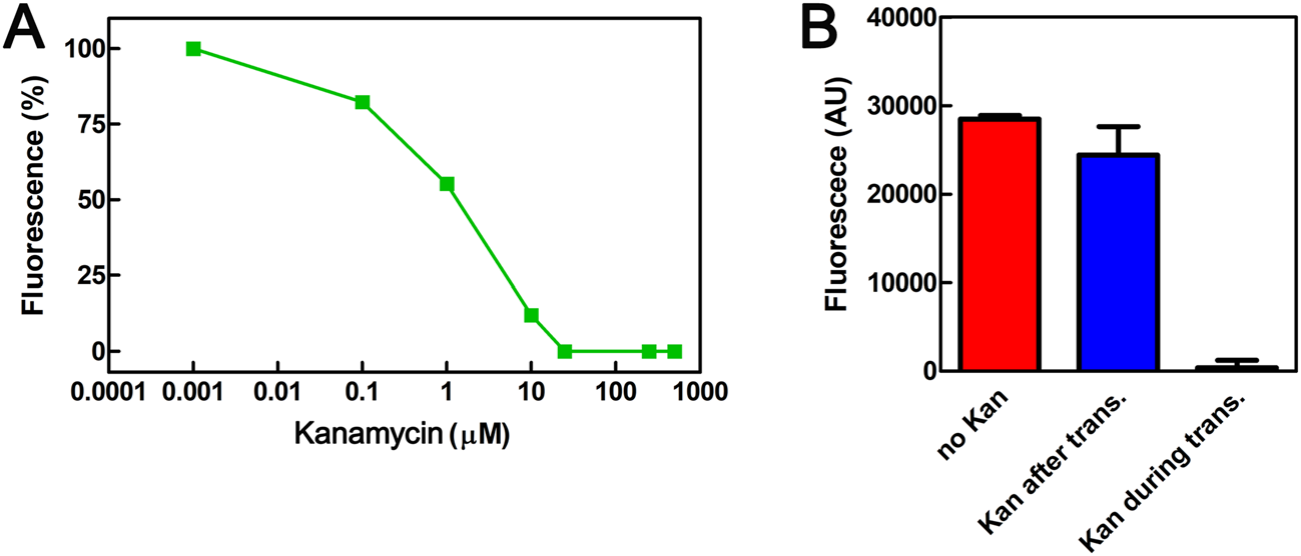
Blocking the synthesis of CSPA:GFP11 uncouples the complementation system. (**A**) In vitro translation of *cspA:gfp11* mRNA (60 pmol) was performed in the presence of increasing concentrations of Kanamycin at 37 °C for 30 min. At the end of incubation, 800 pmol of MBP:GFP1-10_fast_ were added to each reaction tube, which were further incubated at 25 °C for 2 h before measuring fluorescence. (**B**) Fluorescence signal measured when 800 pmol of MBP:GFP1-10_fast_ were added to: (i) sample translated in the absence of antibiotic (red), (ii) sample translated in the absence of antibiotic with simultaneous addition of 50 µM Kanamycin and MBP:GFP1-10_fast_ (blue), (iii) sample translated in the presence of 50 µM Kanamycin (black). The interaction between the two GFP fragments was conducted at 25 °C for 2 h. The error bars represent the standard error calculated from triplicates. In all experiments, the fluorescence signal displayed by control tubes that did not contain mRNA was subtracted.

The addition of MBP:GFP1-10_fast_ is essential for the development of fluorescence (Fig. 6B, white bar), but the synthesis of CSPA:GFP11 is equally important, as demonstrated in the experiment shown in Fig. 8A. In this case, translation was carried out in the presence of increasing concentrations of the protein synthesis inhibitor Kanamycin (Kan). As the concentration of Kanamycin increased, the fluorescence decreased until it was completely extinguished, demonstrating the dependence of the detection system on the synthesis of the product containing the GFP11 tag. Protein synthesis was also conducted in the absence of the antibiotic, in the presence of 50 µM Kanamycin, and by adding Kanamycin simultaneously with MBP:GFP1-10_fast_, i.e., after translation was completed (Fig. 8B). The result of this experiment demonstrates that an antibiotic like Kanamycin inhibits the synthesis of CSPA:GFP11 but not the complementation process (compare blue bar and black bar). ). By adding kanamycin to the reaction at the end of the 37 °C incubation, protein synthesis was blocked before adding the GFP1-10_fast_ fragment. Since there is no significant difference between this condition (blue bar) and the unblocked reaction (red bar), this experiment establishes that during the incubation at 25 °C, necessary for split GFP complementation, the amount of protein that could continue to be synthesized by the CFPS system does not significantly impact the total measured protein synthesis. Therefore, the increase in signal over time shown in Fig. 6C can be attributed to events leading to full maturation of the complex and not to newly synthesized protein.

### Using FAST with template of increasing lentgh

To verify that the experimental set-up could also work with a commercial kit and with proteins other than CspA, the GFP11 tag was fused in-frame with the coding region of three *E. coli* genes of increasing length, namely *hupB*, *moaB*, and *nadE*. *HupB* encodes the β subunit of the histone-like HU protein^22^; *moaB*, part of the *moaABCDE* operon^23^, encodes for a protein whose physiological role has not yet been discovered; finally, *nadE* encodes the NAD + synthetase, which catalyzes the final step of both de novo biosynthesis and salvage of

**Figure 9:**
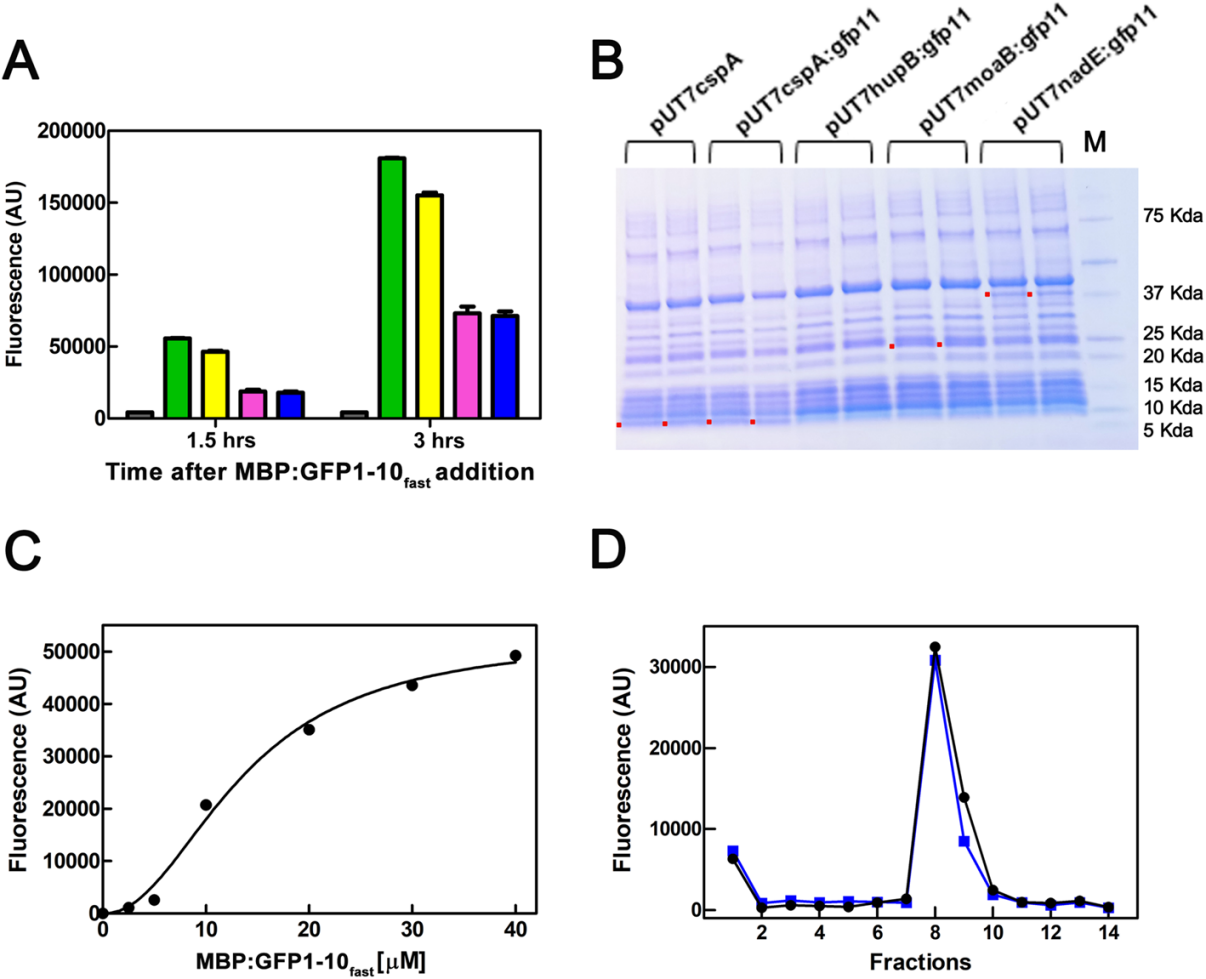
FAST detection of gene:gfp11 fusions of increasing length synthetized using a commercial CFPS. (**A**) Synthesis was performed under the conditions described in Materials and Methods using the PURE express kit. At the end of the incubation time, 400 pmol of MBP:GFP1-10_fast_ were added to each reaction tube, and fluorescence was monitored after 1.5 and 3.5 h. The error bars represent the standard error calculated from triplicates. In all experiments, fluorescence measured in control tubes that did not contain mRNA was subtracted. (**B**) 4–15% PAGE (Mini-PROTEAN TGX BIO-RAD) analysis of 5 µL taken from duplicate samples before the MBP:GFP1-10_fast_ addition. The bands corresponding to the synthetized proteins are indicated with red dots. The full uncropped gel picture is shown in Fig. S11. Note that HU has a certain propensity to form oligomers. However, the denaturing conditions of the electrophoresis reduce this risk. (**C**) Increase in fluorescence resulting from the addition of the indicated concentration of MBP:GFP1-10_fast_ mixed with 5 µL of CFPS reaction carried out with PURE express and pUT7cspA:gfp11. The complementation reaction was performed at 25 °C for 3.5 h. (**D**) Fluorescence emitted by aliquots taken from fractions collected during the purification by affinity chromatography of the MBP:GFP1-10_fast_:GFP11-HupB complex. Fraction 1: flow through; fraction 2: wash; fractions 3–10: elution. The profiles refer to the MBP:GFP1-10_fast_:GFP11-HupB complexe formed in two duplicate reactions as described in Materials and Methods.

NAD +^24^. The constructs for conducting CFPS were generated in the pUT7 plasmid by replacing the coding sequence of *cspA* with the aforementioned three coding regions, while leaving the 5' and 3' regions of *cspA* unchanged. In this way, all the constructs contained the same RBS and transcription terminator (Fig. S10), but produced proteins of gradually increasing sizes, 70 (*cspA*), 90 (*hupB*), 170 (*moaB*), and 275 (*nadE*) amino acids + the GFP11 tag, respectively (Fig. S10). These four DNA constructs, namely pUT7cspA:gfp11, pUT7hupB:gfp11, pUT7moB:gfp11 and pUT7nadE:gfp11, were tested using the PURExpress®(NEB) kit, a coupled in vitro transcription and translation system based on the PUREsystem™^25^ which is a completely purified and reconstituted *E. coli* system. To track protein synthesis, at the end of the incubation, the MBP:GFP1-10_fast_ fragment was added to each reaction tube and fluorescence was recorded. Figure 9A shows that the fluorescent signals developed with all tested constructs and increased over time, as with the in-house prepared crude *E. coli* extracts. However, fluorescence was ~ 2.5 fold lower for the two longer ORFs compared to the two shorter genes. The in vitro synthesis of the five proteins was also analyzed by 4–15% PAGE (Fig. 9B), running 5 µL of the samples before MBP:GFP1-10_fast_ addition. While the bands corresponding to CspA, CspA:gfp11, MoaB and NadE are visible, HupB co-migrates with other protein bands and it is not recognizable. Fluorescence signal obtained with pUT7cspA:gfp11 was very high, being about 6 times more intense than with the in-house prepared *E. coli* extract, probably because of the high efficiency of this coupled transcription/translation system. In fact, as low as 5 µL of the PURE reaction were sufficient to produce an excellent signal, as verified by titrating the reaction with increasing amounts of MBP:GFP1-10_fast_ (Fig. 9C).

In order to evaluate if the MBP:GFP1-10_fast_:GFP11:protein complex resulting from FAST could be purified, a simple protocol was tested exploiting the MBP domain linked to the GFP1-10. An MBP-Trap column (Pharmacia, Sweden) was loaded with a single FAST reaction carried out as described in Materials and Methods and the complex was eluted as for the MBP:GFP1-10 purification. Figure 9D shows that the fluorescent FAST complex binds the amylose resin and can be recovered in the first elution fractions, as expected, with an estimated yield of ~ 50% (based on comparison between initial and recovered fluorescence).

## Discussion

The split GFP system has served as a wellspring of inspiration, spawning myriad applications in molecular and cellular biology across diverse domains^15^. In this study, we have described FAST, a new application of the split-GFP for the detection of protein synthetized in vitro. FAST relies on the fusion of the gene of interest with the C-terminal strand of GFP (GFP11), its transcription and translation in vitro, and the detection of the synthesized product through complementation with an MBP-GFP1-10 fragment developed in this study (Fig. 1). With this approach, we have tracked the synthesis of CspA, HupB, MoaB and NadE (Figs. 6, 8, 9), four proteins of increasing length fused with the GFP11 tag, using laboratory-produced or commercial *E. coli* extracts. Overall, the results demonstrate that FAST is indeed fast, cheap, versatile and practical (Figs. 6, 8, 9) and allows for the quantitative detection (Fig. 6A) of the protein synthesized in CFPS immediately at the end of the reaction and in solution, eliminating the costs associated to radioactive waste disposal or time consuming and demanding procedures like electrophoresis and/or Western blotting. Since the "GFP11 + linker" tag is small in size, it has the advantage of being fused to any gene whose expression has to be monitored in vitro, whether it is a short peptide or a long protein. In this way, the impact of the reporter on expression is significantly reduced compared to the full-length GFP gene.

Since the assay is based on the full synthesis of the protein:GFP11 fusion and its subsequent proper interaction with the MBP-GFP1-10 fragment, the method is particularly suitable for the in vitro screening of factors that may interfere with the translation of a panel of genes. In addition, we proved by biosensor technology that the interaction between MBP:GFP1-10_fast_ and the GFP11 peptide is very stable. Based on this data, we showed that the MBP:GFP1-10_fast_:GFP11:protein complex can not only be detected through fluorescence but can also be purified by exploiting the properties of the MBP protein (Fig. 9D), using the simple, rapid and recognized MBP-based affinity chromatography protocol.

Recently, an article was published in which the split-GFP system was used to monitor enzyme synthesis in CFPS^26^. However, when compared to the protein synthesis detection system described in this work, several significant differences can be outlined. (1) Firstly, the authors used the superfolder GFP variant developed by Cabantous and Waldo^27^, while we evolved the split-GFP system from a different GFP^16^. (2) The GFP used in this work is fused to the N-terminus of MBP. This provides two significant advantages: it avoids the need to purify the GFP1-10 fragment from inclusion bodies^14,26^ as it keeps the protein soluble, and it allows for a simple, rapid, and high-yield purification through affinity chromatography. The GFP1-10 OPT variant, when purified and not fused to other proteins, tends to form unproductive dimers at high concentrations^10^. In this work, we did not conduct experiments to verify the dimer/monomer equilibrium of MBP:GFP1-10s4; therefore, we do not know if fusion with MBP protects the protein from this phenomenon. (3) While the fluorescent signal in our system is high and stabilizes after approximately 4 h, in the system developed by Yuan et al., the fluorescence is rather low in the early hours, to the extent that the authors made their measurements between 8 and 16 h (times when fluorescence had not yet stabilized). (4) Last but not least, the MBP:GFP1-10 fusion allows to purify the MBP:GFP1-10_fast_:GFP11:protein complexes after FAST reaction (Fig. 9D). This is a significant advantage compared to other systems, considering that the complexes are fluorescent, ensuring their traceability even in the case of low protein synthesis.

As for the possible limitations of the system, in our opinion, these could be related to: (A) a potential decrease in fluorescence with increasing sizes of proteins synthesized in vitro due to incomplete synthesis. So far, the longest gene sequence tested with FAST encodes a protein of 303 amino acids (nadE + linker + GFP11 tag). Currently, we cannot establish if there is a limit to the size of the target genes beyond which the synthesis of the C-terminal GFP11 tag, and thus the complementation between the two GFP fragments, is compromised. If such an issue arises, it might be overcome by moving the GFP11 tag from the C-terminus to the N-terminus. (B) Lower sensitivity compared to radioactive labeling, which, as highlighted in Fig. 6A, has a detection power approximately 8–10 times greater than FAST and therefore remains the method of choice for detecting very poorly expressed proteins. (C) Possible signal interference with molecules exhibiting fluorescent properties present in the extracts or libraries to be screened. For this reason, it is always advisable to perform pre-tests to assess the intrinsic fluorescence of the reaction components at the wavelengths to be used. (D) The split-GFP system based on the evolved variant of sfGFP has also been used in vivo in eukaryotic systems^15^. However, the FAST system has only been tested so far with *E. coli* extracts and has never been tested in eukaryotic CFPS systems, where its performance is unknown. Additionally, we did not evaluate if possible post-translational modifications (PTMs) in such system could alter its functionality and performance. Therefore, future research is needed to explore the potential wider applicability of FAST.

It is worth noting that when the protein with the tag is soluble, as in the case of CSPA:GFP11, the complementation test can be performed also in vivo using custom plasmids for the expression of the two GFP fragments (Fig. S8). Therefore, this approach has the potential to enable the comparison of protein expression in vitro and in vivo.

## Conclusions

FAST represents an innovative approach to monitor cell-free protein synthesis, as it possesses the capability to swiftly generate a fluorescent signal which can be detected in solution using a microplate reader. Moreover, this detection process can be seamlessly integrated with the purification of the in vitro-synthesized protein through the use of standardized MBP affinity chromatography. Consequently, FAST could become a valuable tool for screening genes and factors that influence their translation, also in research domains that demand the utilization of CFPS products for subsequent analysis, as in the realms of synthetic biology and protein design.

## Materials and methods

### Bacterial strains

*Escherichia coli BL21(DE3)* + *pLysS*: Genotype;: str. B F^–^ ompT gal dcm lon hsdS_B_(r_B_^–^m_B_^–^) λ(DE3 [lacI lacUV5-T7p07 ind1 sam7 nin5]) [malB^+^]_K-12_(λ^S^) pLysS[T7p20 ori_p15A_](Cm^R^).

*Escherichia coli Stellar (Clontech Laboratories)*. Genotype: F–, endA1, supE44, thi-1, recA1, relA1, gyrA96, phoA, Φ80d lacZΔ M15, Δ (lacZYA—argF) U169, Δ (mrr—hsdRMS—mcrBC), ΔmcrA, λ–.

### Plasmids

pFF-GFP,^16^; pMAL-c5x (New England BioLabs Inc.); pETM11 (EMBL); pUC57cspA:gfp11 (Genewiz); pUT7cspA^4^.

### Molecular cloning of GFP and GFP1-10 into pMAL-c5x

The DNA sequences encoding the full-length GFP or the GFP1-10 fragment were amplified using the forward primer 5'-ACAACCTCGGGATCGAGGGAAGGATGCGTAAAGGAGAAGAACTTTTC-3' and the reverse primer 5'-CCGCCCATGGACATATGTGAAATAGCTTATTAAACTGATGCAGCGTAG-3' for full-length GFP, and the reverse primer 5'-CCGCCCATGGACATATGTGAAATTTATTATGTTCCCTTTTCGTTGGGATCTTTCG-3' for the short fragment. The pFF-GFP plasmid was used as a template, and the high-fidelity *Pfu* DNA polymerase (Fermentas) was used for amplification. The cloning reaction was performed following the In-Fusion protocol (Takara). Subsequently, 2 µl of the reaction mixture were used to transform competent *E. coli* Stellar cells using the CaCl_2_ transformation protocol recommended by Takara.

### Analysis of soluble and insoluble fractions

50 mL of LB + Ampicillin (Amp) were inoculated with 5 mL of saturated culture of *E. coli* Stellar containing the pMAL:GFP or pMAL:GFP1-10 constructs. When the bacterial culture reached an OD_600_ ≈ 0.4, isopropyl-beta-D-1-thiogalactopyranoside (IPTG) was added to a final concentration of 0.3 mM, and the cells were incubated at 37 °C for 2 h. Cells were collected by centrifugation, resuspended in 1 mL of 20 mM Tris–HCl pH 7.5, 200 mM NaCl, 6 mM β-mercaptoethanol, and frozen at − 80 °C. After thawing, the cells were sonicated to disrupt the membranes, and the lysate was briefly centrifuged to remove intact cells. Finally, an extensive centrifugation was carried out at 4 °C to separate the soluble fraction (supernatant) from the insoluble fraction (pellet).

### Purification of MBP:GFP, MBP:GFP1-10 and MBP:GFP1-10_fast_

The overproduction of the proteins was induced by adding 0.3 mM IPTG to *E. coli* Stellar cells harboring corresponding plasmids grown in LB + Amp (60 µg/mL) at 37 °C until reaching an OD_600_ ≈ 0.4. After 3 h of IPTG induction, the cells were centrifuged, and the pellet was resuspended in 12 mL of Column Buffer (20 mM Tris–HCl pH 7.5, 200 mM NaCl, 1 mM EDTA) and frozen at − 80 °C. After thawing, 0.2 mM Benzamidine, 0.2 mM PMSF, and 6 mM β-mercaptoethanol were added to the suspension before cell lysis by sonication. The clarified cellular lysate obtained was diluted with 6 volumes of Column Buffer and loaded onto a chromatography column packed with amylose resin equilibrated with the same buffer. After extensive washing with Column Buffer, the proteins were eluted using Elution buffer (20 mM Tris–HCl pH 7.5, 200 mM NaCl, 1 mM EDTA, 10 mM Maltose, 0.2 mM Benzamidine, 0.2 mM PMSF). Fractions containing the proteins were combined and dialyzed against Storage buffer (20 mM Tris–HCl pH 8.0, 25 mM NaCl, 0.1 mM EDTA, 5% glycerol). The protein concentration was also increased using Amicon ultra-4 3 K devices. At the end of the process, the proteins were frozen at − 80 °C in small aliquots.

### Synthetic GFP11 peptide

The GFP11 peptide (RDHMVLLEFVTAAGIT) was purchased from JPT Peptide Technologies and resuspended in the Storage buffer.

### Molecular cloning of CSPA:GFP11 into pMAL:GFP1-10

The *cspA:gfp11* sequence was amplified using the pUC57 cspA:gfp11 plasmid obtained from Genewiz as a template, the forward primer 5′-ACGAAAAGGGAACATAATAAGGCACACTTAATTATTAAAGGTAATAC-3′, the reverse primer 5′-CCCATGGACATATGTGAAATTTATGTAATCCCAGCAGCAG-3′, and the high-fidelity DNA polymerase *Pfu*. The pMal gfp1-10 plasmid was amplified using the primers 5′-5′TTATTATGTTCCCTTTTCGTTGGGATC-3′ and 9 5′-ATTTCACATATGTCCATGGGCGG-3′ to generate a linear form of the vector suitable for cloning cspA:gfp11 at the desired site. The cloning was performed following the In-fusion protocol, and a portion of the reaction (one-tenth) was used to transform competent *E. coli* Stellar cells.

### Semi-quantitative analysis of CSPA:GFP11 expression level

Samples of *E. coli* Stellar cells, induced or not induced with IPTG, were lysed by boiling in Laemmli sample buffer for 5 min and subjected to 18% SDS-PAGE, followed by transfer onto nitrocellulose membrane in 25 mM Tris, 192 mM glycine, and 1% methanol using the Mini Trans-Blot apparatus (Bio-Rad) for 120 min at 80 V. CSPA:GFP11 was detected essentially as described^7^ using rabbit polyclonal anti-CspA antibodies and secondary antibodies conjugated with peroxidase (Santa Cruz Biotech).

### Site-directed mutagenesis

Site-directed mutations were introduced into the gfp1-10 gene fragment using the forward primer 5′-GGCACAAATTTTCTGTCCGTGGAGAGGGTGAAGG-3′ and the reverse primer 5′-GGATCTTTCGAAAGGACAGTTTGTGTGGACAGGTAATGGTTG-3′, which contained multiple mutation sites compared to the original DNA sequence, inspired by the technique known as TPCR^28^. The mutagenesis reaction was carried out in a final volume of 50 μL, including 40 ng of pMAL:GFP1-10-CSPA:GFP11 plasmid, 20 nM of primers, 200 μM of each dNTP, 1X Pfu Buffer (20 mM Tris–HCl pH 8.8 at 25 °C, 10 mM KCl, 10 mM (NH_4_)_2_SO_4_, 2 mM MgSO_4_, 1.0% Triton X-100, and 1 mg/ml nuclease-free BSA), and 2.0 U of *Pfu* DNA polymerase (Fermentas). The PCR conditions used were as follows: initial denaturation (95 °C, 1 min), followed by 13 cycles of denaturation (95 °C, 30 s), annealing (60 °C, 1 min), and elongation (72 °C, 6.6 min), followed by additional 20 cycles of denaturation (95 °C, 30 s), annealing (67 °C, 1 min), elongation (72 °C, 17.6 min), and a final elongation step of 7 min at 72 °C. At the end of the reaction, 10 U of *Dpn*I were added to 10 μL of the PCR reaction, and after incubation at 37 °C for 2 h, the DNA was directly used to transform *E. coli* Stellar cells made competent by CaCl_2_. Prior to further use, the plasmid was isolated from randomly chosen clones, and the presence of mutations was verified by Sanger DNA sequencing. The resulting mutagenized plasmid was named pMAL:GFP1-10s1-CSPA:GFP11.

### Selection of fluorescent colonies

The selection of fluorescent clones on solid media was performed by growing the cells on LB + Amp (60 µg/mL) agar plates at 37 °C overnight. Subsequently, the plates were transferred to 15 °C for an additional 24 h. Colonies that developed fluorescence during the low-temperature incubation were then streaked onto solid LB medium containing the same antibiotic.

### Random Mutagenesis using “error-prone PCR”

The error-prone PCR (epPCR) reaction was carried out in 100 μL of 10 mM Tris–HCl pH 8.1, 50 mM KCl, 0.5 mM MnCl_2_, 7 mM MgCl_2_, 1 mM dCTP, 1 mM dTTP, 0.2 mM dATP, 0.2 mM dGTP, 0.5 mM forward primer 5′-CAACCTCGGGATCGAGGGAAGG-3′, 0.5 mM reverse primer 5′-GTTCCCTTTTCGTTGGGATCTTTCG-3′, and 0.05 U/µL *Taq* DNA polymerase (Jena Bioscience), using 10 ng of pMAL:GFP1-10s1-CSPA:GFP11 plasmid as a template. The PCR conditions used were as follows: initial denaturation (95 °C, 3 min), followed by 30 cycles of denaturation (94 °C, 30 s), annealing (60 °C, 30 s), and elongation (72 °C, 90 s), and a final elongation step of 2 min at 72 °C. At the end of the reaction, 15 U of *Dpn*I were added to the PCR reaction, and after incubation at 37 °C overnight, the DNA was purified using the PCR cleanup kit (Macherey–Nagel).

### Molecular cloning of randomly mutagenized gfp1-10 fragments into the pMAL-CSPA:GFP11 plasmid.

The plasmid pMAL:GFP1-10-CSPA:GFP11 was amplified using *Pfu* DNA polymerase (Fermentas) with the forward primer 5′-GAAAGATCCCAACGAAAAGGGAAC-3′ and the reverse primer 5′-CCTTCCCTCGATCCCGAGG-3′ to exclude the gfp1-10 region and generate a linear form of the vector suitable for the In-Fusion procedure. After the reaction, the template was removed by digestion with *Dpn*I enzyme. The In-Fusion cloning reaction was carried out according to the specifications in a 10 μL mixture, combining 125 ng of the amplified plasmid pMAL-CSPA:GFP11 and 100 ng of the gfp1-10 amplicon derived from epPCR. After incubation at 50 °C for 15 min, the reaction was diluted with 10 μL of H_2_O. Each time, 4 μL of the diluted reaction were used for the transformation of competent *E. coli* Stellar cells.

### Screening of the mutant library

The fluorescent clones were inoculated in 1 ml of LB + Amp using 12-well Sterilin plates and grown overnight at 37 °C with agitation. The next day, 250 μL of 1:100 dilutions of the overnight cultures were dispensed in triplicates in a 96-well plate (Sterilin) and incubated at 37 °C for 2.5 h. Induction of expression of the mutant GFP fragments was performed by adding IPTG to each well (final concentration 0.3 mM). Immediately after induction, the plate was incubated at 37 °C for 2 h and then transferred to 15 °C for 18 h. After thorough mixing of the samples in the wells, the OD_600_ of each sample was measured using the FLUOstar OMEGA instrument. Then, 200 μL of each sample were taken and transferred to a Black & White 96-well microtiter plate, where fluorescence was measured using the same instrument with excitation and emission wavelengths set at 485 nm and 520 nm, respectively, and gain set to 700. The clone with the highest fluorescence among pMAL:GFP1-10s1-CSPA:GFP11, named pMAL:GFP1-10s2-CSPA:GFP11, was selected, retested to confirm the result, and sequenced. The DNA of GFP1-10s2 was subjected to a second round of random mutagenesis, library preparation, and screening as described above. At the end of this second screening, the two clones with the highest fluorescence, named pMAL:GFP1-10s3-CSPA:GFP11 and pMAL:GFP1-10s4-CSPA:GFP11, were selected, tested in two independent assays to confirm the result, and sequenced.

### Molecular cloning of MBP:GFP1-10_fast_ in the pMAL-c5x vector

The linearized pMAL:GFP1-10_fast_ vector was obtained by PCR using the forward primer 5′-TTATTATGTTCCCTTTTCGTTGGGATC-3′, the reverse primer 5′-ATTTCACATATGTCCATGGGCGG-3′, the high-fidelity Prime STAR GLX DNA polymerase (Takara), and pMAL:GFP1-10_fast_-CSPA:GFP11 as a template to remove the cspA:gfp11 region. At the end of the reaction, the template was digested with *Dpn*I enzyme. 300 ng of linearized plasmid was circularized through overnight ligation at 16 °C in 15 µL of 1X ligation buffer and 2U of DNA ligase. Subsequently, the closed plasmid DNA was used for the transformation of competent *E. coli* Stellar cells using the CaCl_2_ method.

### Molecular cloning of CSPA:GFP11 into pETM11

A linearized form of the pETM11 plasmid suitable for cloning CSPA:GFP11 at the desired site was obtained by PCR using the forward primer 5′-GGCGCCCTGAAAATAAAGATTCTCA-3′, the reverse primer 5′-GCTTGCGGCCGCACTCGA-3′, the high-fidelity PrimeSTAR GLX DNA polymerase, and the pETM11 plasmid as a template. The template was removed after the reaction by *Dpn*I digestion. The cspA:gfp11 insert was amplified using the same DNA polymerase, the forward primer 5′-AGTGCGGCCGCAAGCTTATGTAATCCCAGCAGCTGTTAC-3′, the reverse primer 5′-TATTTTCAGGGCGCCATGTCCGGTAAAATGACTGGTATCG-3′, and the pUC57cspA:gfp11 template. After PCR, both amplicons were purified using the PCR clean-up kit (Sangon Biotech). The cloning procedure was performed following the Infusion protocol, and a portion of the reaction was used for the transformation of competent *E. coli* Stellar cells using the CaCl_2_ method. The plasmid isolated from a selected clone was subsequently used to transform competent *E. coli* BL21(DE3) + pLysS cells. In this case, the selection of transformants was performed on LB + Kan (25 µg/mL) + Cam (20 µg/mL) agar plates.

### Co-induction in *E. coli* of the GFP fragments produced from two different plasmids

Selected clones of *E. coli* BL21(DE3) co-transformed with pETM11-CSPA:GFP11 and pMAL:GFP1-10_fast_ were grown overnight at 37 °C in LB + Kan + Cam medium. 1:100 dilutions of the saturated cultures were grown at 37 °C until reaching an OD600 ≈ 0.4. Under these conditions, IPTG was added to a final concentration of 1 mM to induce protein expression. Before induction, aliquots of each sample were taken as controls. The induced cultures were further incubated at 37 °C, and new aliquots were taken 1, 2, and 3 h after induction, while simultaneously measuring OD_600_ using a spectrophotometer. 200 µL of each sample were used to measure fluorescence using the FLUOstar Omega microplate reader in a Black&White microtiter plate. The size and amount of in vitro synthesized polypetides was analyzed by 15% SDS-PAGE, loading aliquots of the samples taken before and after induction. A similar analysis was conducted also using *E. coli* BL21(DE3) + plysS + pETM11-CSPA:GFP11 or *E. coli* Stellar + pMAL:GFP1-10_fast_ as a control.

### Molecular cloning of *cspA:gfp11*, *hupB:gfp11*, *moaB:gfp11* and *nadE:gfp11* into pUT7cspA plasmid

A linear form of the pUT7cspA plasmid suitable for cloning the linker-gfp11 fragment downstream of the cspA gene was obtained through PCR. The forward primer 5′-TCTCTGCTTAAAAGCACAGAATCTAAG-3′ and the reverse primer 5′-TTTACGATACCAGTCATTTTACCGGAC-3′ were used, along with the high-fidelity

PrimeStar GLX polymerase and the pUT7cspA plasmid^4^ as a template. After the reaction, the template was removed by *Dpn*I digestion. The cspA:gfp11 insert was amplified using the same DNA polymerase, the forward primer 5′-GCTTTTAAGCAGAGATTATGTAATCCCAGCAGCTGTTAC-3′, the reverse primer 5′-GACTGGTATCGTAAAATGGTTCAACGCTGACAAAGGCTTC-3′, and the pUC57cspA:gfp11 plasmid as a template.

For the generation of pUT7hupB:gfp11, pUT7moaB:gfp11, and pUT7nadE:gfp11, the plasmid pUT7cspA:gfp11 was used as a template in a reverse PCR reaction to generate a linear plasmid lacking the *cspA* coding region, named pUT7gfp11. The primers used for this amplification were: 5′-ACCAGCCTGGGCGGTG-3′ and 5′-CATAGTGTATTACCTTTA ATA ATT AAG TG-3′, respectively. At the end of the reaction, the template was removed by *Dpn*I digestion. The *hupB*, *moaB* and *nadE* genes were amplified from *E. coli* chromosome using oligonucleotides with overlaps at the cloning sites in the linear pUT7gfp11.

To amplify *hupB* the forward primer was 5′-AGGTAATACACTATGAATAAATCTCAATTGATCGAC-3′ and the reverse primer was 5′-ACCGCCCAGGCTGGTGTTTACCGCGTCTTTCAG-3′ were used. To amplify *moaB* the forward primer was 5′-AGGTAATACACTATGAGTCAGGTAAGCACTG-3′ and the reverse primer was 5′-ACCGCCCAGGCTGGTTTTCTTCAAATGTGGATG-3′ were used. Finally, to amplify *nadE* the forward primer was 5′-AGGTAATACACTATGACATTGCAACAACAAAT-3′ and the reverse primer was 5′-ACCGCCCAGGCTGGTCTTTTTCCAGAAATCATC-3′ were used.

After PCR, all amplicons were purified using the PCR clean-up kit (Sangon Biotech).

All molecular cloning steps were performed following the In-Fusion protocol. A portion of the reaction mixture was used for the transformation of CaCl_2_-competent *E. coli* Stellar cells.

### In vitro transcription

Templates for in vitro transcription were obtained through PCR using the pUT7cspA:gfp11, pUT7cspA plasmid, pUT7hupB:gfp11, pUT7moaB:gfp11, or pUT7nadE:gfp11, the forward primer 5′-ATGCGTCCGGCGTAGA-3′, which binds upstream of the T7 RNA polymerase promoter, the primer 5′-CGGGATCCAAAATCCCCGCCAAATG-3′, which binds to the 3' UTR region of *cspA*, and the high-fidelity *Pfu* DNA polymerase. Preparative in vitro transcription was conducted as described^7^, and the mRNA was precipitated using LiCl^7^.

### In vitro translation

Crude *E. coli* extracts used in the in vitro protein synthesis system were prepared as described^7^ and subjected to run-off^20^.

The reaction mixture (60 µL) contained 20 mM Tris–HCl (pH 7.7): 12 mM Mg acetate, 80 mM NH_4_Cl, 2 mM DTT, 2 mM ATP, 0.4 mM GTP, 10 mM PEP, 0.025 mg/ml PK, 0.12 mM 10-formyl-tetrahydrofolate, an amino acid mixture containing 0.2 mM of all amino acids (except phenylalanine); a mixture of 0.3 µM [^3^H] phenylalanine and 20 µM non-radioactive phenylalanine (in the radioactive reaction), or 0.2 mM non-radioactive phenylalanine (in the fluorescence detection reaction), 12 µL of S30 run-off, and increasing amounts from 15 to 120 pmol of mRNA. If present, kanamycin antibiotic was added at the concentrations and modes indicated in the text and figure legends. After 30 min of incubation at 37 °C, 20 µL aliquots of each sample were spotted onto 3MM filter discs for radioactivity detection using hot trichloroacetic acid (TCA) precipitation as described^7^. When translation was performed in the absence of radioactive amino acid, at the end of the 37 °C incubation, MBP:GFP1-10_fast_ protein was added in the quantities indicated in the figure and/or legends, and the reaction was further incubated at 25 °C for at least 2 h before transferring 50 µL of each sample into a Black&White microtiter plate for fluorescence measurement using the FLUOstar Omega with gain at 1000 and excitation and emission wavelengths set at 485 nm and 520 nm, respectively.

In vitro translation with the PURExpress kit (NEB) was carried out in 25 µL according to the commercial protocol in the presence of 250 ng of DNA templates (pUT7cspA:gfp11, pUT7hupB:gfp11, pUT7moaB:gfp11 or pUT7nadE:gfp11). The reaction was incubated 4 h at 37 °C. At the end of the translation with the PURExpress kit, 5 µL taken from duplicate samples were analyzed by 4–15% PAGE (Mini-PROTEAN TGX BIO-RAD). The electrophoresis was conducted under denaturing conditions with Tris/Glycine/SDS running buffer using SDS-containing sample buffer. MBP:GFP1-10_fast_ protein was added in the amounts indicated in the figure legends to the remaining 20 µL samples of PURExpress reaction. Samples were further incubated at 25 °C for the indicated times before fluorescence measurement.

### Purification of the MBP:GFP1-10:GFP11:HupB complex by affinity chromatography

HupB:GFP11 was synthetized with the PURE express kit as described above in 13 µL reaction mixtures. At the end of the incubation time, 10 µL of to MBP:GFP1-10_fast_ protein was added (200 pmol) and the complex MBP:GFP1-10_fast_:GFP11:HupB was allowed to form at 25 °C for 3.5 h. Subsequently, Column Buffer was added to each tube to a final volume of 110 µL and the complex was loaded onto a 1 ml MBP-Trap column (Pharmacia, Sweden) equilibrated with the same buffer. After 5 ml washing with Column Buffer, the MBP:GFP1-10_**fast**_:GFP11:HupB complex was eluted using Elution buffer (250 µL /fraction). The recovery of the complex was assessed by measuring the fluorescence of 150 µL aliquots taken from each sample.

### Biosensor binding assay

The interaction between GFP11 and MBP:GFP1-10_fast_ was evaluated according to a standard biosensor assay^29^. Briefly, prior to activation, the carboxylate surface was rinsed with PBS-T (10 mM Na_2_HPO_4_, 2.7 mM KCl, 138 mM NaCl, 0.05% (v/v) Tween-20, pH 7.4), then washed and equilibrated with detergent-free PBS, pH 7.4, to prevent the possible shielding of carboxylic groups. Next, upon activation of carboxylate groups with an equimolar solution of EDC and NHS^30^, MBP:GFP1-10_fast_ was covalently blocked to the surface. Functionalized surface was eventually equilibrated with 20 mM Tris–HCl, 200 mM NaCl, pH 8.0, and MBP:GFP1-10_fast_ was tested for binding at different concentrations of GFP11 in the range of 10–100 nM. The biosensor chamber was maintained at 37 °C throughout the experiment. Raw data were analyzed with mono- and biexponential models with Fast Fit software (Fison Applied Sensor Technology; Affinity Sensors), the validity of each model to fit time courses being assessed by the F-test procedure.

### Supplementary Information

Supplementary Information.

## Data Availability

All data generated and analyzed during this study are included in this published article and its supplementary information files.
