# Supplementary material for: FAST, a method based on split-GFP for the detection in solution of proteins synthesized in cell-free expression systems
Source: Sci Rep. 2024 Apr 5;14:8042. doi: 10.1038/s41598-024-58588-5 (PMC10997616; doi:10.1038/s41598-024-58588-5)
Supplement: Supplementary file 1 — Supplementary Information. [file 41598_2024_58588_MOESM1_ESM.pdf]

Supplementary File

FAST, a method based on split-GFP for the detection in solution of proteins synthesized in cell-free expression systems

Thuy Duong Pham, Chiara Poletti, Therese Manuela Nloh Tientcheu, Massimiliano Cuccioloni, Roberto Spurio, Attilio Fabbretti, Pohl Milon and Anna Maria Giuliodori

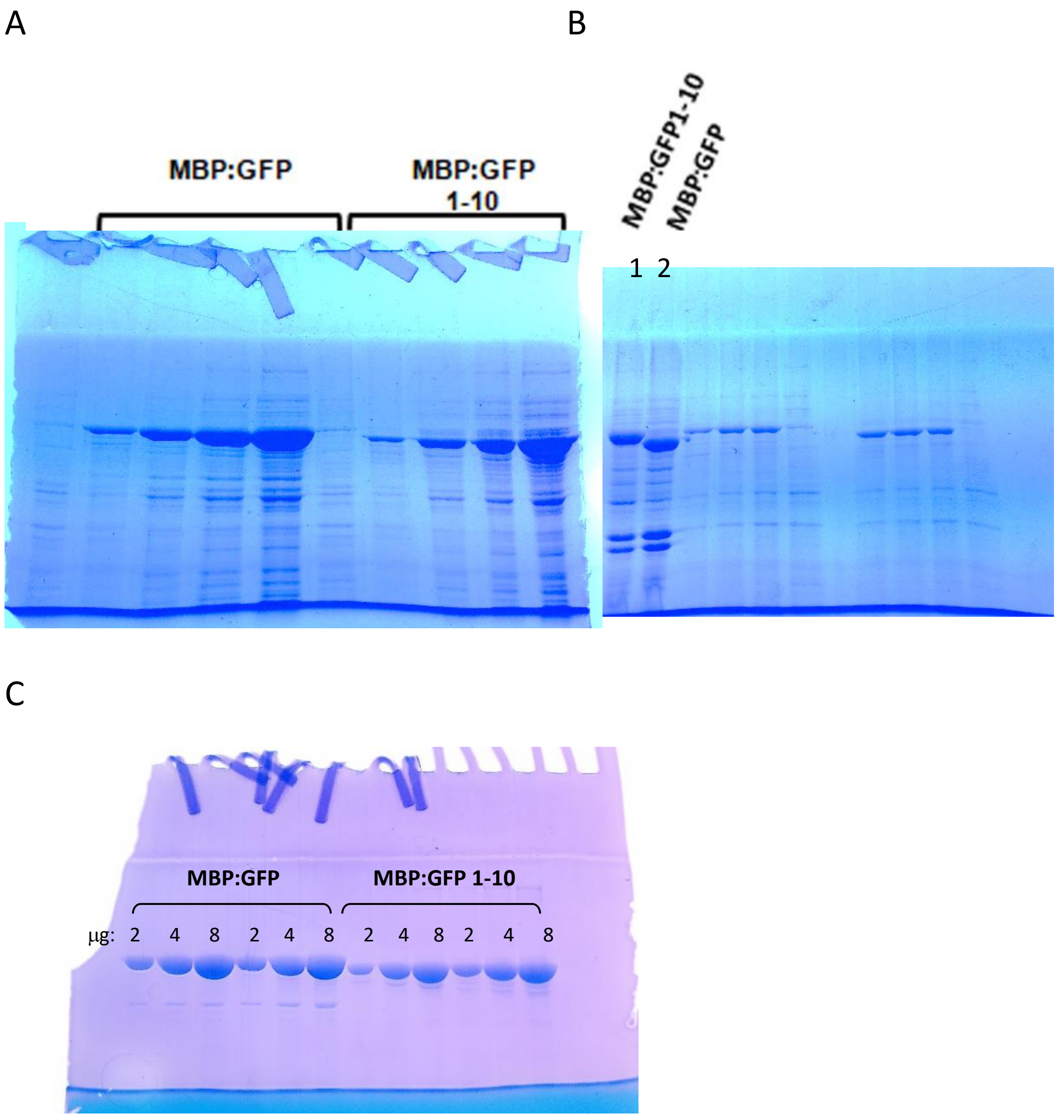

Figure S1. Solubility of MBP:GFP and MBP:GFP1-10. Uncropped gel pictures of the electrophoretic separation by 10% SDS-PAGE of: A) the soluble fraction of *E. coli* Stellar containing MBP:GFP or MBP:GFP1-10; B) lanes 1 and 2 refer to the insoluble fraction presents in the pellet produced after cell disruption and removal of unlysed cells, resuspended in 6M urea. The remaining lanes refer to unrelated samples. C) Uncropped full gel of the electrophoretic separation by 10% SDS-PAGE of increasing amounts of the indicated purified proteins.

1) Nucleotide sequence of CSPA:GFP11
5'-
GGCACACTTAATTATTA**AAGG**TAATACACT**ATG**TCCGGTAAAATGACTGGTATCGTAAAATGGT
TCAACGCTGACAAAGGCTTCGGCTTCATCACTCCTGACGATGGCTCTAAAGATGTGTTCGTAC
ACTTCTCTGCTATCCAGAACGATGGTTACAAATCTCTGGACGAAGGTCAGAAAGTGTCCTTCA
CCATCGAAAGCGGCGCTAAAGGCCCGGCAGCTGGTAACGTAACCAGCCTGGGCGGTGGCTC
TGGTGGCGGTTCTATGCGTGACCACATGGACCTTCTTGAGTTTGTAACAGCTGCTGGGATTAC
ATAA-3'

2) Amino acid sequence of CSPA:GFP11
MSGKMTGIVKWFNADKGFGFITPDDGSKDVFVHFSAIQNDGYKSLDEGQKVSFTIESGAKGPAAG
NVTSLGGGSGGGSMRDHMDLLEFVTAAGIT

3) MBP:**GFP1-10**
MKIEEGKLVIWINGDKGYNGLAEVGKKFEKDTGIKVTVEHPDKLEEKFPQVAATGDGPDIIFWAHD
RFGGYAQSGLLAEITPDKAFQDKLYPFTWDAVRYNGKLIAYPIAVEALSLIYNKDLLPNPPKTWEEI
PALDKELKAKGKSALMFNLQEPYFTWPLIAADGGYAFKYENGKYDIKDVGVDNAGAKAGLTFLVD
LIKNKHMNADTDYSIAEAAFNKGETAMTINGPWAWSNIDTSKVNYGVTVLPTFKGQPSKPFVGVLS
AGINAASPNKELAKEFLENYLLTDEGLEAVNKDKPLGAVALKSYEEELVKDPRIAATMENAQKGEI
MPNIPQMSAFWYAVRTAVINAASGRQTVDEALKDAQTNSSSNNNNNNNNNNLGIEGR**MRKGEEL**
FTGVVPILVELDGDVNGHKFSVSGEGEGDATNGKLTLKFICTTGKLPVPWPTLVTTLTYGVQCFA
RYPDHMKQHDFFKSAMPEGYVQERTISFKDDGTYKTRAEVKFEGDTLVNRIELKGIDFKEDGNIL
GHKLEYNFNSHNVYITADKQKNGIKANFKIRHNVEDGSVQLADHYQQNTPIGDGPVLLPDNHYLS
TQSALSKDPNEKGT

4) MBP:**GFP1-10s1**
MKIEEGKLVIWINGDKGYNGLAEVGKKFEKDTGIKVTVEHPDKLEEKFPQVAATGDGPDIIFWAHD
RFGGYAQSGLLAEITPDKAFQDKLYPFTWDAVRYNGKLIAYPIAVEALSLIYNKDLLPNPPKTWEEI
PALDKELKAKGKSALMFNLQEPYFTWPLIAADGGYAFKYENGKYDIKDVGVDNAGAKAGLTFLVD
LIKNKHMNADTDYSIAEAAFNKGETAMTINGPWAWSNIDTSKVNYGVTVLPTFKGQPSKPFVGVLS
AGINAASPNKELAKEFLENYLLTDEGLEAVNKDKPLGAVALKSYEEELVKDPRIAATMENAQKGEI
MPNIPQMSAFWYAVRTAVINAASGRQTVDEALKDAQTNSSSNNNNNNNNNNLGIEGR**MRKGEEL**
FTGVVPILVELDGDVNGHKFSVRGEGEGDATNGKLTLKFICTTGKLPVPWPTLVTTLTYGVQCFA
RYPDHMKQHDFFKSAMPEGYVQERTISFKDDGTYKTRAEVKFEGDTLVNRIELKGIDFKEDGNIL
GHKLEYNFNSHNVYITADKQKNGIKANFKIRHNVEDGSVQLADHYQQNTPIGDGPVLLPDNHYLS
TQTVLSKDPNEKGT

Figure S2. 1) Sequence of the CSPA:GFP11 fusion in pMAL vector. The RBS of the *cspA* gene (light blue) controls the translation of the fusion in the bicistronic construct cloned into the pMAL vector. The coding region of the cspA gene (blue) is cloned upstream of the linker (gray) preceding the GFP11 fragment sequence (green). The SD sequence is indicated in bold, and the mutated nucleotides are shown in red. 2) Expression of the fusion produces the protein with the indicated sequence. The different colors correspond to different gene regions. The modified amino acid in GFP11 is shown in red. Amino acid sequences of MBP:GFP1-10 (3) and MBP:GFP1-10s1 (4). The amino acids highlighted in yellow are those changed by site-directed mutagenesis in MBP:GFP1-10s1 with respect to MBP:GFP1-10 . The sequences in bold correspond to GFP1-10 and GFP1-10s1 sequences, respectively.

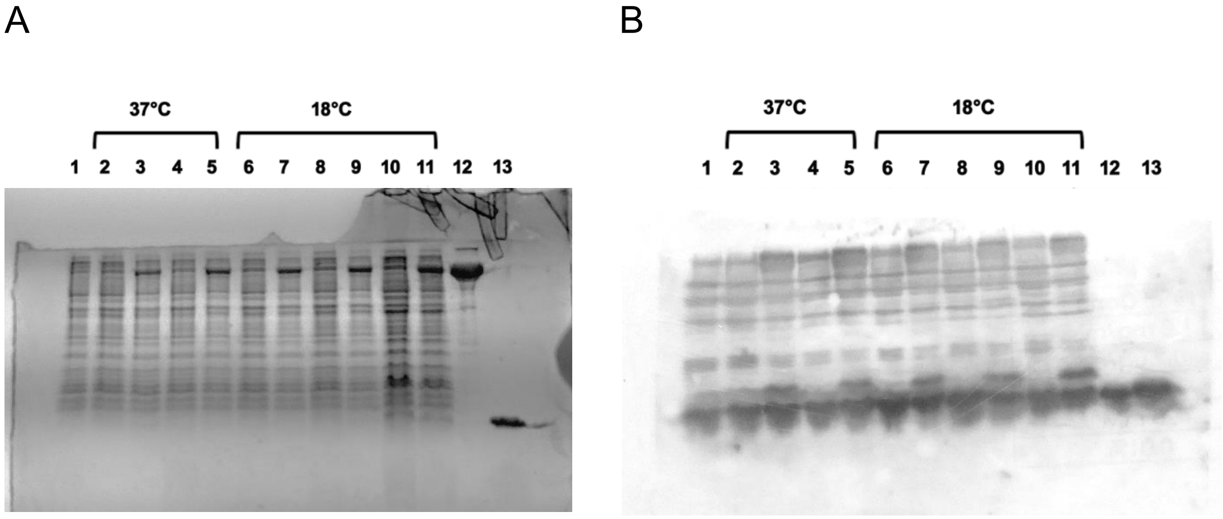

Figure S3. Full uncropped pictures of: A) MBP:GFP1-10 expression monitored by 18% SDS-PAGE and B) CSPA:GFP11 expression by Western blot. Protein synthesis was induced in *E. coli* Stellar cells containing the bicistronic construct. The conditions are those described in the main text, Fig. 3.

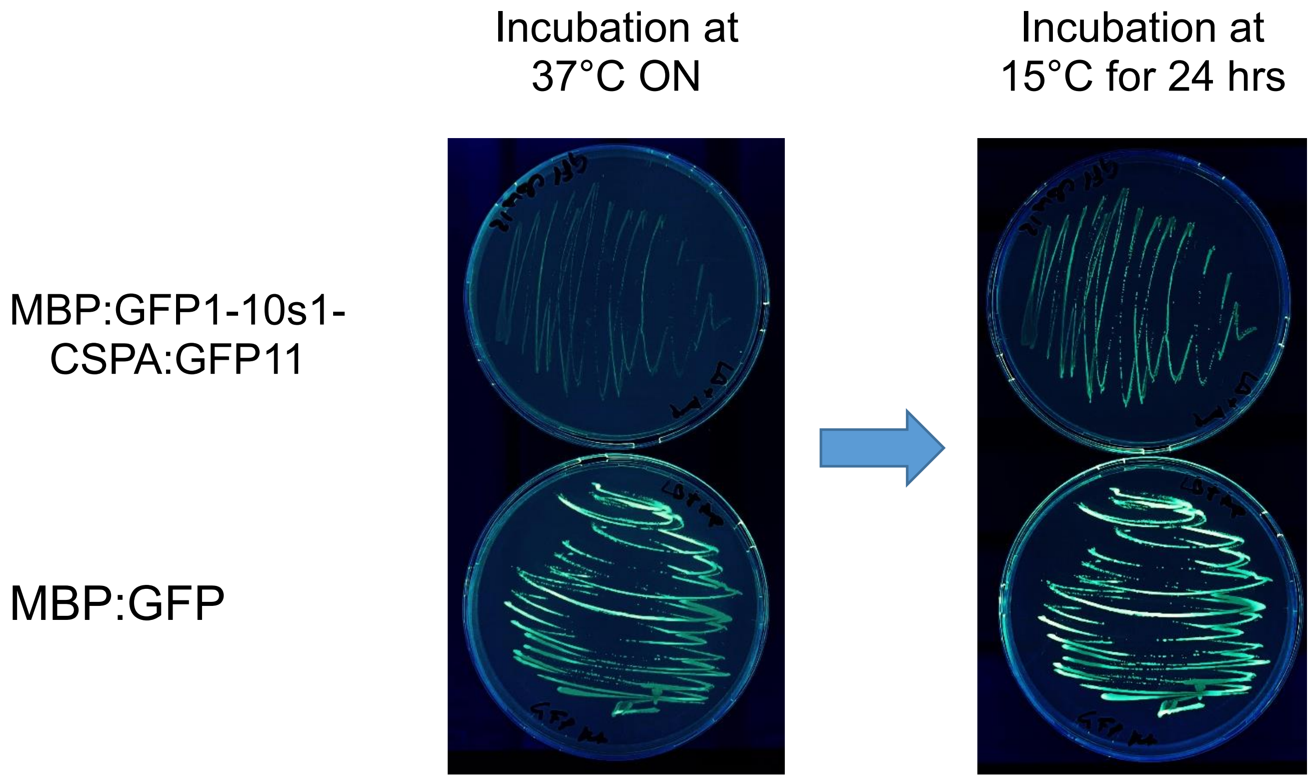

Figure S4. ***E. coli*** **Stellar cells transformed with pMAL:GFP or pMAL:GFP1-10s1-CSPA:GFP11.** Fluorescence of the cells on LB+Amp plates (60 µg/ml) is observed after overnight incubation at 37°C only with the pMAL:GFP construct, while with the pMAL:GFP1-10s1-CSPA:GFP11 construct (and its subsequent derivatives), an additional incubation for approximately 24 hours at temperatures ≤ 20°C is required.

1) MBP:**GFP1-10s2.**

MKIEEGKLVIWINGDKGYNGLAEVGKKFEKDTGIKVTVEHPDKLEEKFPQVAATGDGPDIIFWAHD
RFGGYAQSGLLAEITPDKAFQDKLYPFTWDAVRYNGKLIAYPIAVEALSLIYNKDLLPNPPKTWEEI
PALDKELKAKGKSALMFNLQEPYFTWPLIAADGGYAFKYENGKYDIKDVGVDNAGAKAGLTFLVD
LIKNKHMNADTDYSIAEAAFNKGETAMTINGPWAWSNIDTSKVNYGVTVLPTFKGQPSKPFVGVLS
AGINAASPNKELAKEFLENYLLTDEGLEAVNKDKPLGAVALKSYEEELVKDPRIAATMENAQKGEI
MPNIPQMSAFWYAVRTAVINAASGRQTVDEALKDAQTNSSSNNNNNNNNNNLGIEGR**MRKGEEL
FTGVVPILVELDGDVNGHKFSVRGEGEGDATNGKLTLKFICTTGKLPVPWPTLVTTLTYGVQCFA
RYPDHMKQHDFFKSAMPEGYVQERTISFKDDGTYKTRAEVKFEGDTLVNRIELKGIDFKEDGNIL
GHKLEYNFNSHKVYITADKQKNGIKANFKIRHNVEDGSVQLADHYQQNTPIGDGPVLLPDNHYLS
TQTVLSKDPNEKGT**

2) MBP:**GFP1-10s3.**

MKIEEGKLVIWINGDKGYNGLAEVGKKFEKDTGIKVTVEHPDKLEEKFPQVAATGDGPDIIFWAHD
RFGGYAQSGLLAEITPDKAFQDKLYPFTWDAVRYNGKLIAYPIAVEALSLIYNKDLLPNPPKTWEEI
PALDKELKAKGKSALMFNLQEPYFTWPLIAADGGYAFKYENGKYDIKDVGVDNAGAKAGLTFLVD
LIKNKHMNADTDYSIAEAAFNKGETAMTINGPWAWSNIDTSKVNYGVTVLPTFKGQPSKPFVGVLS
AGINAASPNKELAKEFLENYLLTDEGLEAVNKDKPLGAVALKSYEEELVKDPRIAATMENAQKGEI
MPNIPQMSAFWYAVRTAVINAASGRQTVDEALKDAQTNSSSNNNNNNNNNNLGIEGR**MRKGEEL
FTGVVPILVELDGDVNGHKFSVRGEGEGDATNGKLTLKFICTTGKLPVPWPTLVTTLTYGVQCFA
RYPDHMKQHDFFKSAMPEGYVQERTISFKDDGTYKTRAEVKFEGDTLVNRIELKGIDFKEDGNIL
GHKLEYNFNSHKVCITADKQKNGIKANFKIRHNVEDGSVQLADHYQQNTPIGDGPVLLPDNHYL
STQTVLSKDPNEKGT**

3) MBP:**GFP1-10s4 (**MBP:**GFP1-10$_{fast}$).**

MKIEEGKLVIWINGDKGYNGLAEVGKKFEKDTGIKVTVEHPDKLEEKFPQVAATGDGPDIIFWAHD
RFGGYAQSGLLAEITPDKAFQDKLYPFTWDAVRYNGKLIAYPIAVEALSLIYNKDLLPNPPKTWEEI
PALDKELKAKGKSALMFNLQEPYFTWPLIAADGGYAFKYENGKYDIKDVGVDNAGAKAGLTFLVD
LIKNKHMNADTDYSIAEAAFNKGETAMTINGPWAWSNIDTSKVNYGVTVLPTFKGQPSKPFVGVLS
AGINAASPNKELAKEFLENYLLTDEGLEAVNKDKPLGAVALKSYEEELVKDPRIAATMENAQKGEI
MPNIPQMSAFWYAVRTAVINAASGRQTVDEALKDAQTNSSSNNNNNNNNNNLGIEGR**MRKGEEL
FTGVVPILVELDGDVNGHKFSVRGEGEGDATNGKLTLKFICTTGKLPVPWPTLVTTLTYGVQCFA
RYPDHMKQHDFFKSAMPEGYVQERTISFKDDGTYKTRAEVKFEGDTLVNRIELKGIDFKEDGNIL
GHKLEYNFNSHKVYITADKQKNGIKANFKIRHNVEDGSVQLADHYQQNTPIGDGPVLLPDNHYLS
TQTVLSKDPNEKGT**

Figure S5. Amino acid sequences of MBP:GFP1-10s2 (1), MBP:GFP1-10s3 (2), MBP:GFP1-10s4 sequence (3). The lysine amino acid highlighted in green is mutated compared to the MBP:GFP1-10 and MBP:GFP1-10s1 sequences, while the cysteine highlighted in cyan is mutated compared to the MBP:GFP1-10, MBP:GFP1-10s1, and MBP:GFP1-10s2 sequences. The amino acid sequence of MBP:GFP1-10s4(fast) is identical to MBP:GFP1-10s2.

1) ***gfp1-10s3*.**

5′-

ATGCGTAAAGGAGAAGAACTTTTCACTGGAGTTGTCCCAATTCTTGTTGAATTAGATGG
TGATGTTAACGGGCACAAATTTTCTGTCCGTGGAGAGGGTGAAGGTGATGCAACAAAC
GGAAAACTTACCCTTAAATTTATTTGCACTACTGGAAAACTACCTGTTCCATGGCCAAC
ACTTGTCACTACTTTGACTTATGGTGTTCAATGCTTTGCGAGATACCCAGATCATATGAA
ACAGCATGACTTTTTCAAGAGTGCCATGCCCGAAGGTTATGTACAGGAAAGAACTATAT
CATTCAAAGATGACGGGACCTACAAGACACGTGCTGAAGTCAAGTTTGAAGGTGATAC
TCTTGTTAATAGAATCGAGTTAAAAGGTATTGATTTTAAAGAAGATGGAAACATTCTTGG
ACACAAATTGGAATACAACTTTAACTCACACAAAGTAT**G**CATCACGGCAGACAAACAAA
AGAATGGAATCAAAGCTAACTTCAAAATTAGACACAACGTTGAAGATGGAAGCGTTCAA
CTAGCAGACCATTATCAACAAAATACTCCAATTGGCGATGGCCCTGTCCTATTACCAGA
CAACCATTACCTGTCCACACAAACTGTCCTTTCGAAAGATCCCAACGAAAAGGGAACA
TAA-3’

2) ***gfp1-10s4(fast)*.**

5’-

ATGCGTAAAGGAGAAGAACTTTTCACTGGAGTTGTCCCAATTCTTGTTGAATTAGATGG
TGATGTTAACGGGCACAAATTTTCTGTCCGTGGAGAGGGTGAAGGTGATGCAACAAAC
GGAAAACTTACCCTTAAATTTATTTGCACTACTGGAAAACTACCTGTTCCATGGCCAAC
ACTTGTCACTACTTTGACTTATGGTGTTCAATGCTTTGCGAGATACCCAGATCATATGAA
ACAGCATGACTTTTTCAAGAGTGCCATGCCCGAAGGTTATGTACAGGAAAGAACTATAT
CATTCAAAGATGACGGGACCTACAAGACACGTGCTGAAGTCAAGTTTGAAGGTGATAC
TCTTGTTAATAGAATCGAGTTAAAAGGTATTGATTTTAAAGAAGATGGAAACATTCTTGG
ACACAAATTGGAATACAACTTTAACTCACACAAAGT**T**TACATCACGGCAGACAAACAAA
AGAATGGAATCAAAGCTAACTTCAAAATTAGACACAACGTTGAAGATGGAAGCGTTCAA
CTAGCAGACCATTATCAACAAAATACTCCAATTGGCGATGGCCCTGTCCTATTACCAGA
CAACCATTACCTGTCCACACAAACTGTCCTTTCGAAAGATCCCAACGAAAAGGGAACA
TAA-3’

Figure S6. Nucleotide sequences of gfp1-10s3 (1) and gfp1-10s4 (2). The bases highlighted in red are those mutated compared to gfp1-10s2 and gfp1-10s1. The figure does not show the sequence of the *malE* gene.

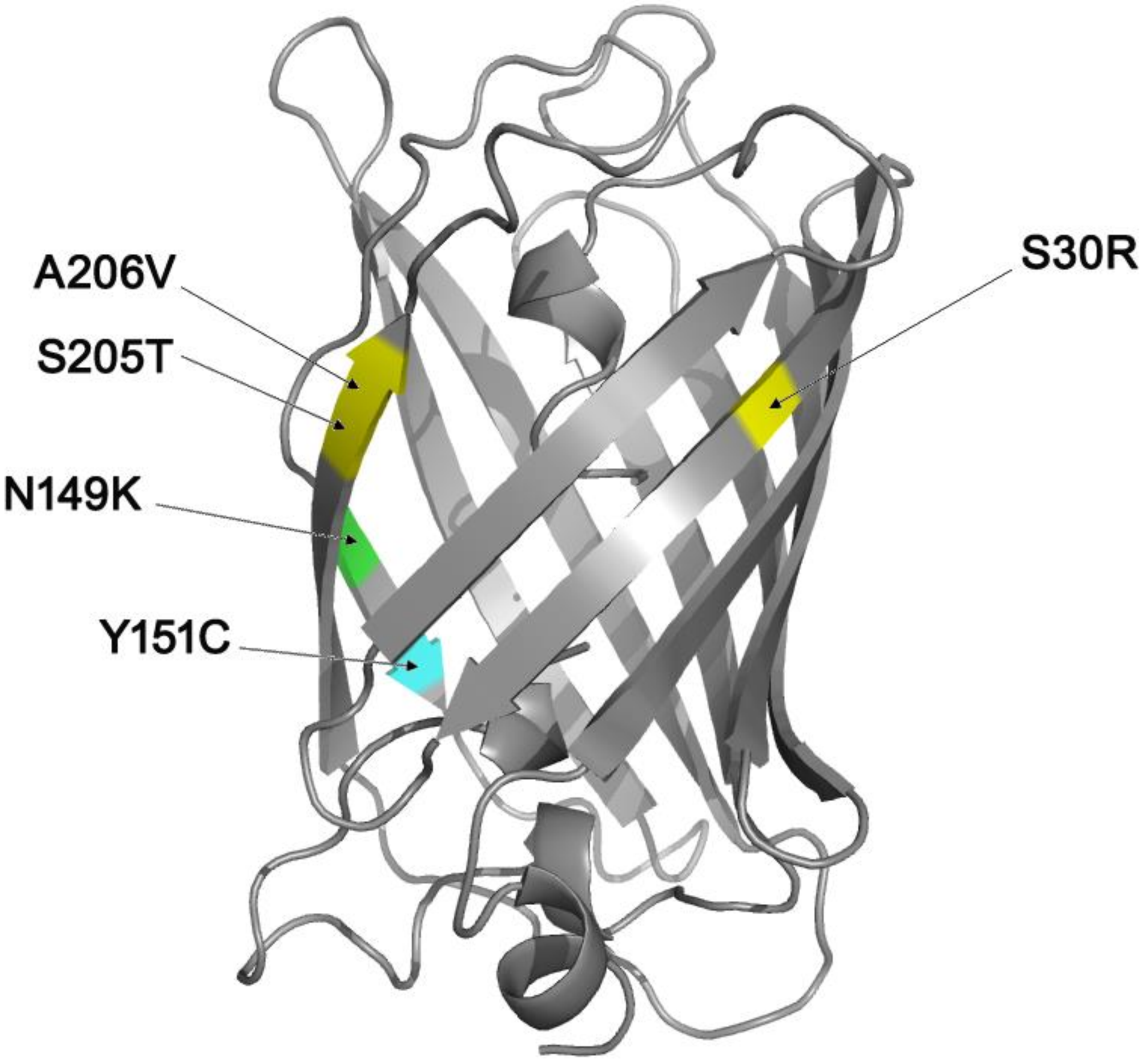

Figure S7. 3D model of GFP1-10 showing the localization of selected mutations in the MBP:GFP1-10s2, s3, and s4(fast) variants.

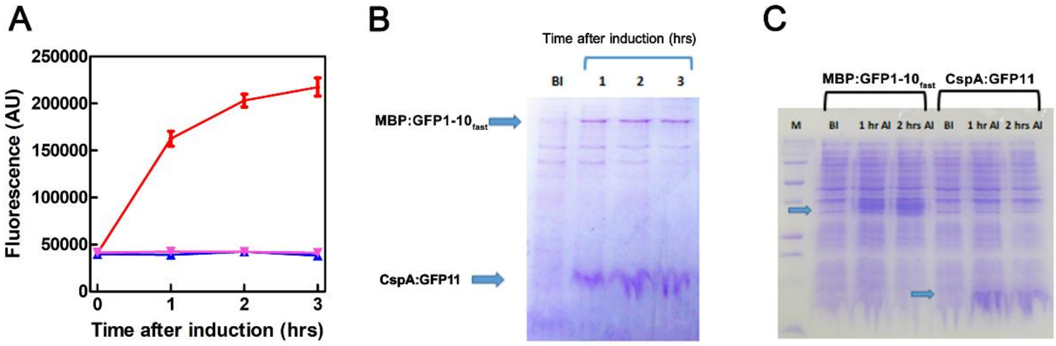

Figure S8. **Co-induction of MBP:GFP1-10$_{\mathbf{fast}}$ and CSPA:GFP11**. A) Fluorescence signal measured over time after IPTG induction in: *E. coli* BL21(DE3) cells co-transformed with the pETM11-CSPA:GFP11 and pMAL:GFP1-10$_{\mathbf{fast}}$ plasmids (red trace); *E. coli* BL21(DE3)+ pLysS transformed with the pETM11-CSPA:GFP11 plasmid (purple trace); *E. coli* Stellar transformed with the pMAL:GFP1-10$_{\mathbf{fast}}$ (blue trace). Error bars indicate the standard deviation calculated from the fluorescence emitted by 4 different clones. B) Verification of the simultaneous overexpression of MBP:GFP1-10$_{\mathbf{fast}}$ and CSPA:GFP11 in clone 1 by 18% PAGE-SDS. C) Verification of the separate overexpression of MBP:GFP1-10$_{\mathbf{fast}}$ or CSPA:GFP11 by 18% PAGE-SDS in either *E. coli* Stellar or *E. coli* BL21(DE3)+ pLysS, respectively. The bands corresponding to the respective proteins are indicated by an arrow. The full uncropped gel pictures are shown in Fig. S9.

A

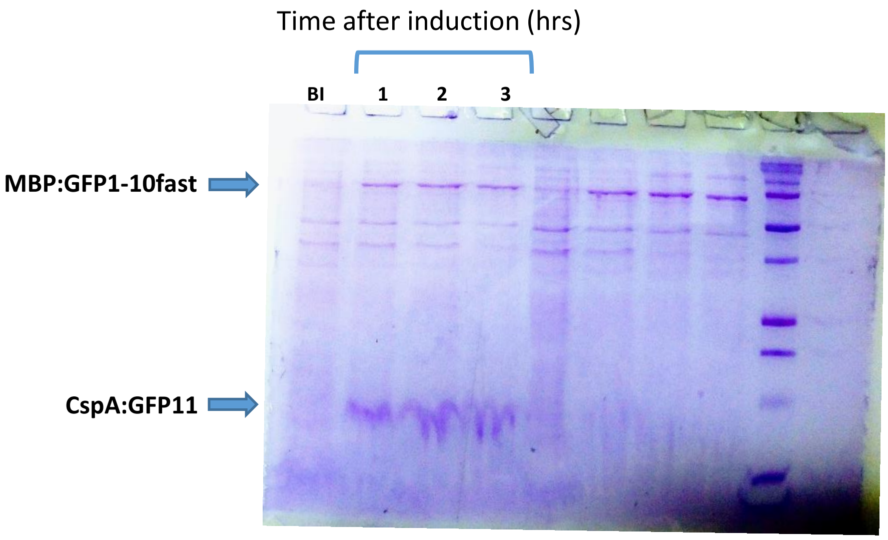

B

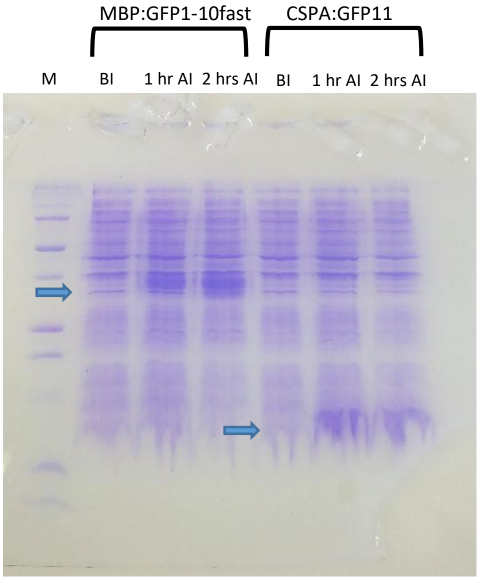

Figure S9. A) Uncropped gel picture of the simultaneous overexpression of MBP:GFP1-10$_{\mathbf{fast}}$ and CSPA:GFP11 by 18% PAGE-SDS (lanes 1-4). The samples loaded in the following lanes refer to an experiment not discussed in the text. B) Uncropped gel picture of the separate overexpression of MBP:GFP1-10$_{\mathbf{fast}}$ or CSPA:GFP11 by 18% PAGE-SDS in either *E. coli* Stellar or *E. coli* BL21(DE3)+ pLysS, respectively. The bands corresponding to the respective proteins are indicated by an arrow.

cspA:gfp11

5’-
AACGGTTTGACGTACAGACCATTAAAGCAGTGTAGTAAGGCAAGTCCCTTCAAGAGTT
ATCGTTGATACCCCTCGTAGTGCACATTCCTTTAACGCTTCAAAATCTGTAAAGCACG
CCATATCGCCGAAAGGCACACTTAATTATTAAAGGTAATACACT**ATG**TCCGGTAAAATG
ACTGGTATCGTAAAATGGTTCAACGCTGACAAAGGCTTCGGCTTCATCACTCCTGACG
ATGGCTCTAAAGATGTGTTCGTACACTTCTCTGCTATCCAGAACGATGGTTACAAATCT
CTGGACGAAGGTCAGAAAGTGTCCTTCACCATCGAAAGCGGCGCTAAAGGCCCGGC
AGCTGGTAACGTAACCAGCCTGGGCGGTGGCTCTGGTGGCGGTTCTATGCGTGACCA
CATGGACCTTCTTGAGTTTGTAACAGCTGCTGGGATTACA**TAA**TCTCTGCTTAAAAGCA
CAGAATCTAAGATCCCTGCCATTTGGCGGGGATTTTTTT-3’

MSGKMTGIVKWFNADKGFGFITPDDGSKDVFVHFSAIQNDGYKSLDEGQKVSFTIESGAK
GPAAGNVTSLGGGSGGGSMRDHMDLLEFVTAAGIT

hupB:gfp11

5’-
AACGGTTTGACGTACAGACCATTAAAGCAGTGTAGTAAGGCAAGTCCCTTCAAGAGTT
ATCGTTGATACCCCTCGTAGTGCACATTCCTTTAACGCTTCAAAATCTGTAAAGCACG
CCATATCGCCGAAAGGCACACTTAATTATTAAAGGTAATACACT**ATG**AATAAATCTCAA
TTGATCGACAAGATTGCTGCAGGGGCTGATATCTCTAAAGCTGCGGCTGGCCGTGCG
TTAGATGCTATTATTGCTTCCGTAACTGAATCTCTGAAAGAAGGGGATGATGTAGCAC
TGGTAGGTTTTGGTACTTTTGCCGTTAAAGAGCGTGCTGCCCGTACTGGCCGCAACC
CGCAGACCGGTAAAGAGATCACCATCGCTGCTGCTAAAGTACCGAGCTTCCGTGCAG
GTAAAGCACTGAAAGACGCGGTAAACACCAGCCTGGGCGGTGGCTCTGGTGGCGGT
TCTATGCGTGACCACATGGACCTTCTTGAGTTTGTAACAGCTGCTGGGATTACA**TAA**T
CTCTGCTTAAAAGCACAGAATCTAAGATCCCTGCCATTTGGCGGGGATTTTTTT-3’

MNKSQLIDKIAAGADISKAAAGRALDAIIASVTESLKEGDDVALVGFGTFAVKERAARTGR
NPQTGKEITIAAAKVPSFRAGKALKDAVNTSLGGGSGGGSMRDHMDLLEFVTAAGIT

moaB:gfp11

5’-
AACGGTTTGACGTACAGACCATTAAAGCAGTGTAGTAAGGCAAGTCCCTTCAAGAGTT
ATCGTTGATACCCCTCGTAGTGCACATTCCTTTAACGCTTCAAAATCTGTAAAGCACG
CCATATCGCCGAAAGGCACACTTAATTATTAAAGGTAATACACT**ATG**AGTCAGGTAAG
CACTGAATTTATCCCGACCCGTATTGCTATTCTTACGGTTTCTAATCGTCGCGGTGAA
GAAGACGATACCTCCGGTCACTATCTGCGCGATTCGGCGCAAGAAGCGGGCCATCA
CGTTGTCGATAAAGCCATTGTGAAAGAAAACCGCTACGCTATTCGCGCTCAGGTATCT
GCGTGGATCGCCAGCGACGATGTACAAGTGGTATTGATTACGGGTGGTACTGGCCT

GACGGAAGGTGATCAGGCTCCCGAAGCATTGCTGCCGTTGTTCGACCGTGAAGTTGA
AGGTTTTGGTGAAGTGTTCCGGATGTTGTCGTTTGAAGAGATTGGCACTTCCACGTTG
CAATCTCGTGCGGTAGCGGGCGTCGCCAACAAAACGCTGATTTTCGCCATGCCGGG
TTCGACCAAAGCGTGCCGTACCGCATGGGAAAATATCATCGCGCCGCAGCTGGATG
CCCGTACGCGTCCGTGTAATTTCCATCCACATTTGAAGAAAACCAGCCTGGGCGGTG
GCTCTGGTGGCGGTTCTATGCGTGACCACATGGACCTTCTTGAGTTTGTAACAGCTG
CTGGGATTACA**TAA**TCTCTGCTTAAAAGCACAGAATCTAAGATCCCTGCCATTTGGCG
GGGATTTTTTT-3’

MSQVSTEFIPTRIAILTVSNRRGEEDDTSGHYLRDSAQEAGHHVVDKAIVKENRYAIRAQV
SAWIASDDVQVVLITGGTGLTEGDQAPEALLPLFDREVEGFGEVFRMLSFEEIGTSTLQS
RAVAGVANKTLIFAMPGSTKACRTAWENIIAPQLDARTRPCNFHPHLKKTSLGGGSGGGS
MRDHMDLLEFVTAAGIT

nadE:gfp11

5’-
AACGGTTTGACGTACAGACCATTAAAGCAGTGTAGTAAGGCAAGTCCCTTCAAGAGTT
ATCGTTGATACCCCTCGTAGTGCACATTCCTTTAACGCTTCAAAATCTGTAAAGCACG
CCATATCGCCGAAAGGCACACTTAATTATTAAAGGTAATACACT**ATG**ACATTGCAACA
ACAAATAATAAAGGCGCTGGGCGCAAAACCGCAGATTAATGCTGAAGAGGAAATTCG
TCGTAGTGTCGATTTTCTGAAAAGCTACCTGCAAACTTATCCGTTCATTAAATCACTGG
TGCTCGGGATCAGCGGCGGTCAGGACTCCACGCTTGCCGGAAAGCTGTGCCAGATG
GCGATTAATGAGCTGCGCCTGGAAACCGGCAACGAATCACTGCAATTTATTGCCGTA
CGCCTGCCCTATGGTGTTCAGGCCGACGAACAAGATTGCCAGGATGCCATTGCCTTT
ATTCAACCGGATCGCGTATTAACCGTTAATATCAAGGGCGCGGTATTGGCCAGCGAA
CAGGCATTGCGGGAAGCAGGCATTGAACTGAGCGATTTTGTCCGTGGCAATGAAAAA
GCGCGTGAGCGGATGAAAGCACAATATAGCATTGCGGGTATGACCAGCGGTGTCGT
GGTGGGCACCGATCATGCAGCAGAAGCCATTACCGGATTCTTCACTAAATATGGTGA
CGGCGGTACGGACATTAATCCGCTGTATCGTCTCAACAAACGTCAGGGTAAACAGTT
ACTGGCGGCATTAGCTTGCCCGGAACACCTTTATAAGAAAGCGCCAACGGCCGATCT
GGAAGATGATCGCCCTTCTCTGCCAGATGAAGTGGCACTCGGCGTGACCTATGACAA
TATCGACGACTATCTGGAAGGGAAAAACGTACCTCAACAGGTCGCCAGAACAATAGA
GAACTGGTATCTGAAAACCGAACATAAACGCCGTCCGCCAATTACCGTTTTCGATGAT
TTCTGGAAAAAGACCAGCCTGGGCGGTGGCTCTGGTGGCGGTTCTATGCGTGACCA
CATGGACCTTCTTGAGTTTGTAACAGCTGCTGGGATTACA**TAA**TCTCTGCTTAAAAGC
ACAGAATCTAAGATCCCTGCCATTTGGCGGGGATTTTTTT-3’

MTLQQQIIKALGAKPQINAEEEIRRSVDFLKSYLQTYPFIKSLVLGISGGQDSTLAGKLCQM
AINELRLETGNESLQFIAVRLPYGVQADEQDCQDAIAFIQPDRVLTVNIKGAVLASEQALRE
AGIELSDFVRGNEKARERMKAQYSIAGMTSGVVVGTDHAAEAITGFFTKYGDGGTDINPL
YRLNKRQGKQLLAALACPEHLYKKAPTADLEDDRPSLPDEVALGVTYDNIDDYLEGKNVP
QQVARTIENWYLKTEHKRRPPITVFDDFWKKTSLGGGSGGGSMRDHMDLLEFVTAAGIT

Figure S10. Sequences of the gfp11-tagged genes cloned in pUT7 plasmid. The 5' and 3' UTR of the *cspA* gene are indicated in light blue. The SD sequence is underlined, while the initiation and stop codons are in bold. The coding region of the gene of interest (*cspA*, *hupB, moaB* and *NadE*) is cloned upstream of the linker (gray) preceding the GFP11 fragment sequence (green). The amino acid sequence of each gene:gfp11 fusion is shown below the corresponding nucleotide sequence, using the same color code.

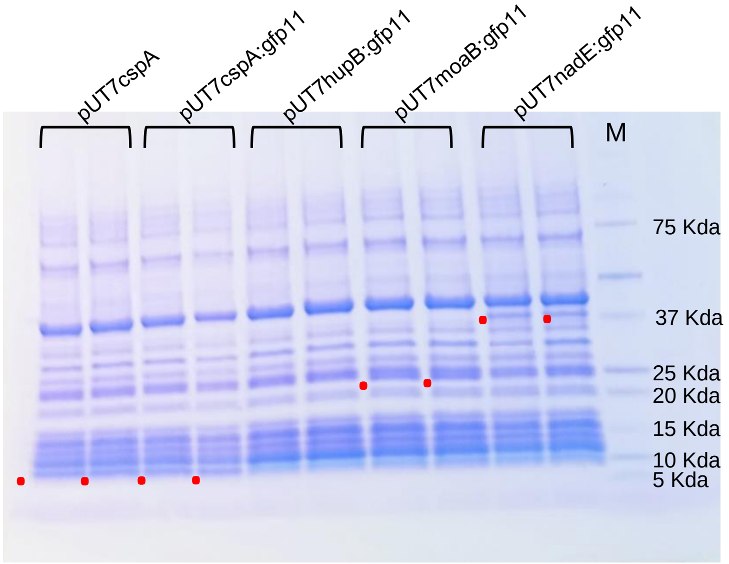

Figure S11. Uncropped gel picture of the 4-15% PAGE (Mini-PROTEAN TGX BIO-RAD) analysis of 5 µL of translation reaction taken from duplicate samples before the MBP:GFP1-10$_{\mathbf{fast}}$ addition. See Fig. 9 of the main text for further details.
